# Lime amendment to chronically acidified forest soils results in shifts in prokaryotic communities

**DOI:** 10.1128/aem.02171-24

**Published:** 2025-12-29

**Authors:** Maggie Hosmer, Robyn J. Wright, Caitlin McCavour, Kevin Keys, Shannon Sterling, Morgan G. I. Langille, John Rohde

**Affiliations:** 1 Department of Microbiology and Immunology, Dalhousie University 152982, Halifax, Canada; 2 Department of Pharmacology, Dalhousie University 55967, Halifax, Canada; 3 Department of Earth and Environmental Sciences, Dalhousie University 152954, Halifax, Canada; 4 Department of Lands and Forestry, Government of Nova Scotia, Halifax, Nova Scotia, Canada; Universidad de los Andes, Bogotá, Colombia

**Keywords:** 16S rRNA amplicon sequencing, microbiome, lime, chronic acidification

## Abstract

A consequence of past acid rain events has been chronic acidification of Nova Scotian forests, leading to a loss of essential nutrients and subsequent decreases in forest productivity and biodiversity. Liming—supplementing forests with crushed limestone—can restore essential nutrients to acidified soils and increase the pH of soils and the carbon capture by forests through the promotion of tree growth. Liming treatments are often assessed through tree growth measurements, although little is known about how microorganisms respond to these changes in pH and nutrient availability. Understanding the impacts of liming on microbial communities will help determine whether liming is a good remediation strategy for Nova Scotia. A pilot study evaluating liming in acidified forests in Nova Scotia began in 2017. Microbiome analysis (prokaryotic 16S and fungal ITS2 rRNA gene amplicon sequencing) of three different horizons (depths; upper forest floor, lower forest floor, and upper B horizon) of soil in a softwood forest area showed significant differences between lime-treated and control soils for the prokaryotic but not fungal communities, particularly in the uppermost soil horizon. Several genera from the Alphaproteobacteria class were significantly higher in abundance in treated than control soils, whereas genera from the Acidobacteriia (previously Acidobacteriae) class were significantly lower in abundance in treated versus control soils. Soil chemistry analysis of the same three horizons showed a significant increase in base cations and pH of the uppermost soil horizon in control versus treatment sites.

**IMPORTANCE:**

Forests are increasingly being managed with an emphasis on understanding how forests function. Lime amendments are used to promote forest health and increase resilience to climate change. To date, only a handful of studies have analyzed the effects of liming on microbial communities in forest soils. Our study combines soil chemistry with prokaryotic and fungal communities of limed and control soils. Shifts in microbial composition that are coincident with liming may provide early indications of the effectiveness of liming and provide insight into the roles of microbes in forest health.

## INTRODUCTION

Forests are under multiple threats due to anthropogenically driven global climate change; these include increasing temperatures, drought, increased pests, and increased severity and frequency of fires (1). Forests are the dominant land cover in Canada and are a key foundation of the Canadian economy. Canadian forests sustain biodiversity and regulate biogeochemical cycling. This ecosystem is habitat to thousands of species and stores of 10%–30% of global terrestrial carbon (2). There is an increasing need to understand how forests function and develop strategies and practices to increase forest resilience and their use as natural climate solutions (3). Bacteria, archaea, fungi, and viruses play important roles in biogeochemical cycling, and their contributions to forest health are becoming increasingly appreciated. Bacteria and fungi break down forest biomass to recycle nutrients, promote soil formation, and assist trees in acquiring nutrients (4, 5). Viruses are abundant in forest soils, although their roles in soil health are poorly understood. Similarly, archaea are abundant in some forest soils and may contribute to nitrification, although nitrification by bacteria appears to dominate (4, 6).

Decades of acidic emissions from fossil fuel burning have resulted in significant acid rain deposition over much of northeastern North America. Legislation, most notably the Clean Air Act Amendments of 1990, drastically reduced industrial emissions and resulted in a rebound of many affected surface waters. By contrast, soils have been much slower to recover. Nova Scotian forests remain compromised by decades of acidification because of their unique geology (slow weathering, base cation-poor bedrock, and soil parent materials) and extensive wetlands (7, 8). Even prior to the chronic acidification due to acid rain, softwood forests are known to be acidic, particularly in Nova Scotia (9, 10); nevertheless, the acid rain resulted in even greater chronic acidification. Many Nova Scotian soils have now become depleted in base cations such as calcium (Ca^2+^) and magnesium (Mg^2+^) that are essential for productive forests (11). In addition, chronic acidification has resulted in the increased release of toxic forms of aluminum in soils and waterbodies across the province (7, 8). One potentially effective method to restore lost productivity in Nova Scotian forests is to replace depleted nutrients and alkalinity through liming. Liming, as defined in this study, is the application of crushed limestone (either calcite [CaCO_3_] or dolomite [CaMgCO_3_]) to soil and has been used for many years to promote forest ecosystem recovery in Scandinavian countries that have also suffered chronic terrestrial acidification (12).

After surface application, limestone slowly dissolves in soils, releasing bicarbonate and base cations (13). Bicarbonate raises the pH of soils, and base cations become available for uptake by plants and microbes. Raising pH also causes aluminum (Al^3+^) to more favorably interact and complex with organic molecules, making it less available for leaching and uptake by plants (14, 15). The effects of liming are most commonly assessed through measurements of soil chemistry (16), but they can also be measured through changes in forest health. In most cases, liming results in increased productivity of acidified forests, and two recent studies in Canada have shown that liming increases tree health for decades after treatment has been applied (17, 18). To date, only a handful of studies have analyzed the microbial response to liming in forest soils. Moreover, these studies are difficult to compare because they differ in region, amendment type, application methodology, and dominant tree species. It is difficult to identify commonalities that may exist in changes in microbial community structure in limed and un-limed forest soils (19–22). To examine the effects of liming in an acidic softwood forest site in Nova Scotia, we have characterized soil chemistry and used 16S rRNA gene and Internal Transcribed Spacer (ITS) 2 rRNA gene amplicon sequencing. This approach has allowed us to analyze the effects of liming on alterations in forest soil microbiome composition in the context of a comprehensive analysis of soil chemistry, producing a rich data set. We initially examined the overall community composition of prokaryotes and fungi and then identified specific taxa that are associated with liming treatment.

## MATERIALS AND METHODS

### Study site

Otter Ponds Demonstration Forest (Mooseland, Nova Scotia) is the location of an experiment to evaluate the effectiveness of helicopter liming (application of crushed dolomite) as a remediation strategy for acidified forests. A predominantly mature red spruce (*Picea rubens*) site, divided into separate control and treatment sections, was previously chosen based upon uniformity and accessibility. Both sections contained five measurement plots with a radius of 10.3 m and a center point determined with ArcGIS (Table S1). Ten tons per hectare (10 tons/ha = 1,000 g/m^2^) of crushed dolomitic limestone (Mosher Limestone, 8433 Hwy 224, Upper Musquodoboit, NS B0N 2M0) was applied to the treatment section using helicopters in October 2018.

### Soil sampling

**Fig 1:**
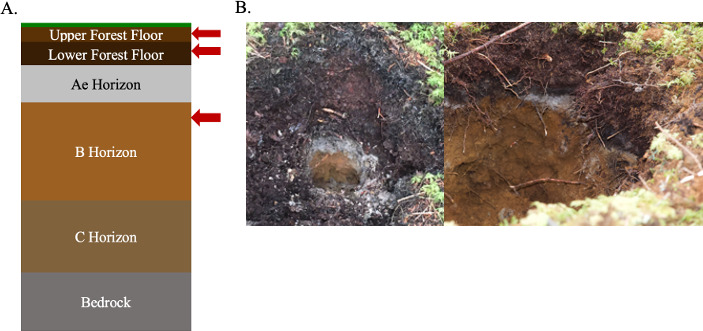
General Podzolic soil horizon schematic for each plot with a representative soil photo. (**A**) General schematic of typical soil horizons. Red arrows indicate the horizons sampled. Typically, the upper forest floor is ~1–5 cm depth, the lower forest floor is ~5–10 cm depth, and the upper B horizon is ~20–35 cm depth. The Ae horizon, C horizon, and bedrock were not sampled. (**B**) Representative soil pit showing forest floor and mineral soil horizons. The upper forest floor is being below the moss ground cover, the lower forest floor is being directly above the white/gray Ae layer, and the upper B horizon is being directly below the Ae layer.

Soils in the study areas are classified as well-drained Orthic Humo-Ferric Podzols, derived from glacial till high in quartzite (10). Forest floor horizons were field-classified as Hemimors in which F and H horizons are >2 cm, fungal mycelia are dominant, decaying wood content is low, and F horizons are >50% of forest floor thickness (23). Three soil horizons were sampled: upper forest floor (~1–5 cm depth: F horizon), lower forest floor (~5–10 cm: F/H transition), and upper B horizon (~20–35 cm depth from the mineral soil surface) (Fig. 1). One round of pre-treatment and three rounds of post-treatment sampling were completed. In the pre-treatment round (October 2018), one sample of each horizon per plot was taken for analysis. In post-treatment round one (October 2019), three samples of each horizon per plot were taken for soil chemistry analysis. The difference in sample sizes was due to the assumption that there would be less variability pre-treatment, whereas post-treatment variability in lime application could lead to higher between-sample variability. In post-treatment rounds two (October 2020) and three (July 2021), two and three samples, respectively, of each horizon per plot were collected for microbiome analysis, resulting in a total of 150 soil samples from all three rounds. To help address within-plot variability, random bearings from the plot center were used to select the first sample location within each plot, with locations of subsequent samples chosen by going 180° (round two) or 120° (round three) from the original random bearing. Approximately 500 g of soil was collected per sample and placed in sterile plastic bags. Bags were transported on ice to Dalhousie University, Nova Scotia, for further analysis. All samples slated for microbiome analysis were stored at −80°C until analyzed.

### Soil chemistry measurements

Base cations and available metals were analyzed using inductively coupled plasma mass spectrometry (ICP-MS) by AGAT Laboratories (Dartmouth N.S.) as described by the Standard Methods for the Examination of Water and Wastewater (24). Briefly, soil samples were weighed and digested with hydrochloric acid and nitric acid. After digestion, the samples were cooled and brought to volume with double deionized water and analyzed by ICP-MS. Soil samples were analyzed for total C, N, and S by the Laurentian Forestry Centre in Quebec City, Quebec, using a Laboratory Equipment Corporation induction furnace according to the manufacturer’s manual.

### DNA extraction, library preparation, and amplicon sequencing

DNA was isolated from soil samples using the Qiagen PowerSoil DNA Isolation Kit following the manufacturer’s protocol except for one modification: samples were held on the vortex by hand versus using a vortex adapter as described in the protocol. The isolated DNA was stored at −20°C until further analysis. Extracted DNA samples were sent to the Integrated Microbiome Resource (Dalhousie University) where library preparation and Illumina MiSeq amplicon sequencing (MiSeq Reagent Kit v3) were completed. The V4–V5 regions of the prokaryotic 16S rRNA gene were amplified using primers 515FB (5'–GTGYCAGCMGCCGCGGTAA) and 926R (5'–CCGYCAATTYMTTTRAGTTT) for bacterial/archaeal identification (25, 26). The ITS2 region of the fungal ribosomal genes was amplified using primers ITS86(F) (5'–GTGAATCATCGAATCTTTGAA) and ITS4(R) (5'–TCCTCCGCTTATTGATATGC) for fungal identification (27). Library preparation and amplicon sequencing followed the standard operating procedures set out in Comeau et al. (28).

### Sequencing data processing

Processing of the sequencing data followed the Microbiome Helper workflow (28), utilizing QIIME 2 (29). Briefly, raw reads were imported into QIIME 2 (version 2021.8), and primers were trimmed using Cutadapt (30). Reads were denoised using the DADA2 (31) denoising algorithm (two errors were allowed for each of the forward and reverse reads) with trim lengths of 275/180 bp and 260/200 bp for forward/reverse reads from 16S rRNA or ITS2 genes, respectively. The resulting amplicon sequence variants (ASVs) were classified using the SILVA v138 database (32) for 16S rRNA gene amplicons for bacterial/archaeal identification and the UNITE v8 database (33) for ITS2 rRNA gene amplicons for fungal identification. For 16S rRNA (hereafter 16S) and ITS2 rRNA (hereafter ITS2) gene amplicons, sequences that were not classified at the phylum level or were present at below 10 total reads were removed from further analysis. For 16S, sequences that belonged to mitochondria or chloroplasts were also removed. A phylogenetic tree was built using SEPP (34) with a reference phylogeny created using the SILVA reference database (version 128) (32) for 16S gene sequences.

### Statistical analysis

Downstream analyses were carried out using custom R (version 2021.09.0) and Python (version 3.8.8) scripts. Differences in soil chemistry measurements between pre- and post-treatment were assessed using paired *t*-tests and between control and treatment samples using unpaired *t*-tests within the Python package Scipy (35). Maps with soil chemistry values were plotted onto a map obtained from ArcGIS using the Python package matplotlib (36). Alpha diversity was assessed using rarefied tables with Faith’s phylogenetic diversity for 16S and Simpson’s diversity, Chao 1 richness, and Shannon’s diversity for both 16S and ITS2. Analysis of variance (ANOVA) with post-hoc Tukey’s HSD tests for differences between groups was carried out using the Python package bioinfokit (37). Beta diversity was assessed using relative abundances and weighted UniFrac distance (38, 39) for 16S or Bray-Curtis dissimilarity (40, 41) for ITS2, calculated within the R package phyloseq (42) with visualization using a principal coordinate analysis (PCoA) with the Python package scikit-bio (43). Normalization for the PCoA plots was done using the Python package deicode (44). Associations between microbial composition and liming as well as soil horizon, site, sample within site, and sampling round were assessed using PERMANOVA tests within the R package vegan (45). False discovery rate (FDR) (Benjamini-Hochberg methods) adjustments were also done on the resulting *P*-values from PERMANOVA testing. The dispersion or variance within groups (i.e*.,* treatment or soil horizon) was assessed using the “betadisper” function, and significance was determined using ANOVA, both from the vegan R package (45).

In order to assess whether there exist any taxa that were differentially abundant between the control and treatment samples, we utilized the following three differential abundance tools: ALDEx (46) (version ALDEx2 1.30.0), ANCOM (47) (version ANCOM-BC 2.0.3), and MaAsLin (48) (version MaAsLin2 1.12.0), which use normalizations of centered log ratio, additive log ratio, and rarefied abundance, respectively. These tools were chosen based on the recommendations of Nearing & Douglas et al. (49), although we note that the versions we used of these tools were different from those used by Nearing & Douglas et al. We therefore report on how many, and which, of the tools identify a given taxon as differentially abundant—those with a Benjamini-Hochberg adjusted *P*-value of ≤ 0.1 in ALDEx, a q-value of ≤ 0.1 in ANCOM, or a q-value of ≤ 0.1 in MaAsLin—and consider a taxon to be differentially abundant if it is identified by at least two of the three tools. We ran the tests for differential abundance using treatment as well as sampling round for all three tools, and additionally for the interaction between treatment and sampling round where the tools had this functionality (for ANCOM and ALDEx). Figures used the additional Python packages matplotlib (36), tidyr (50), numpy (51), scipy (35), pandas (52), Biopython (53), and ete3 (54).

## RESULTS

### Lime amendment was effective for altering soil chemistry

**Fig 2:**
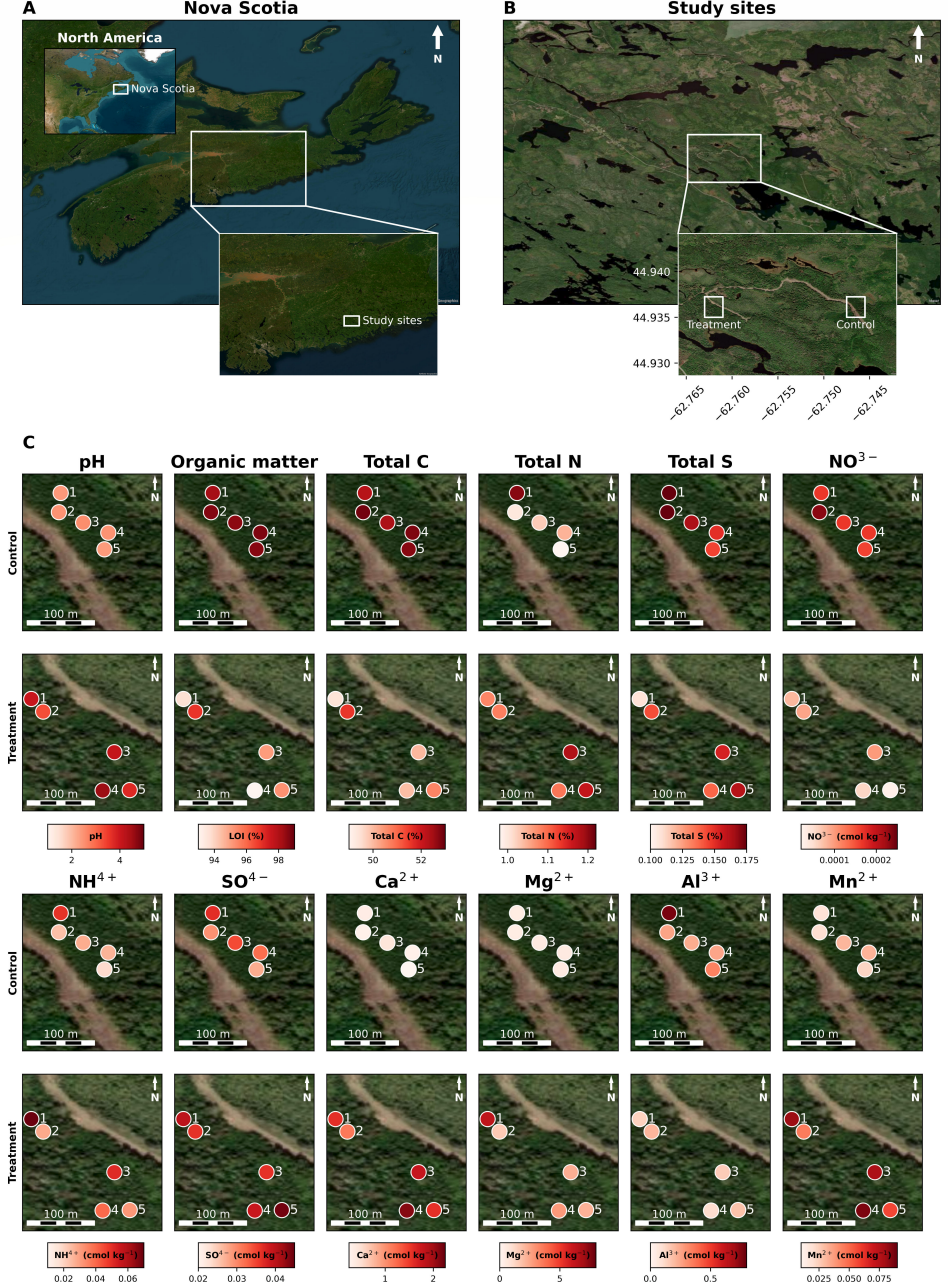
Map of sample sites with soil chemistry data. (**A**) Map showing the study location within Nova Scotia. (**B**) Map showing the areas used for each of the control (**C**) and treatment (T) sections. (**C**) Maps of five sampling plots each are shown in the control and treatment sites/sections. Each set of two panels shows soil chemistry data (pH, organic matter [LOI], total C, total N, total S, NO^3-^, NH^4+^, SO^4-^, Ca^2^, Mg^2+^, Al^3+^, or Mn^2+^) for the upper forest floor for five control (top) and five treatment (bottom) plots. Points marking each plot are colored by mean soil chemistry values post-treatment (see Table S1 for details; Fig. S1 to S3 show pre- and post-treatment soil chemistry measurements, the change in soil chemistry measurements pre- and post-treatment, and the differences between control and treatment plots post-treatment, respectively. Each control panel shows longitudes −62.7475 to −62.745 (x-axis; left to right) and latitudes 44.9375 to 44.9345 (y-axis; top to bottom). Each treatment panel shows longitudes −62.7630 to −62.7605 (x-axis; left to right) and latitudes 44.9375 to 44.9345 (y-axis; top to bottom).

**Fig 3:**
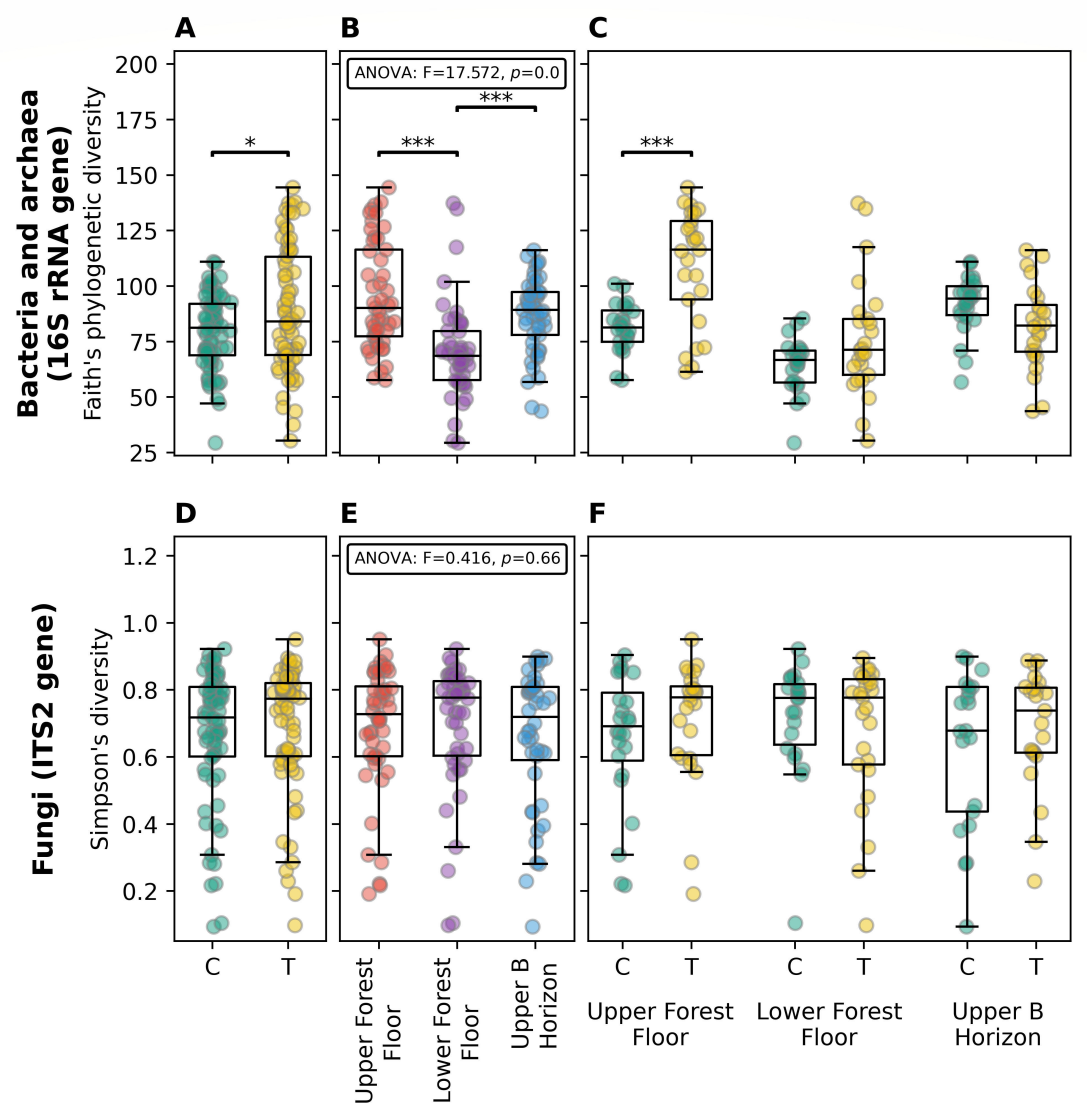
Alpha diversity of microbial community samples. The results for the 16S rRNA gene community (bacteria and archaea) are shown in the top panels (**A–C**), with the ITS2 rRNA gene community (fungi) shown in the bottom panels (**D-E**). Plots show alpha diversity within samples grouped by: control (**C**) and treatment (T) in panels A and D; by soil horizon in panels B and (**E**); and by control and treatment for each soil horizon in panels (**C** and **F**). In each of these panels, sample data are shown as individual points, whereas boxes show the median and upper and lower quartiles, and whiskers show the range of data (1.5 times the interquartile range). Significant differences between treatment and control results (ANOVA) are shown using asterisks, with * denoting *P* ≤ 0.05 and *** *P* ≤ 0.005.

The treatment section had a target of 10 tons/ha (1,000 g/m^2^) of crushed lime dropped from a helicopter. The amount of crushed lime that was estimated to have been applied at each plot varied between 330 and 1,220 g/m^2^ (means of 330, 770, 543, 1,220, and 510 g/m^2^ were measured for plots 1–5, respectively), reflecting heterogeneity in the application method. Soil chemistry measurements (pH, organic matter [LOI] total C, total N, total S, nitrate [NO^3-^], ammonium [NH^4+^], sulfate [SO^4-^], calcium [Ca^2+^], aluminum [Al^3+^], magnesium [Mg^2+^], and manganese [Mn^2+^]) were taken pre-treatment and 1 year post-treatment (1 year before the first microbiome samples) (Table S1). Although we have compared the pre- and post-treatment measurements to assess the effectiveness of the liming (Supp. Results; Fig. S1 and S2), we focus here on the post-treatment soil chemistry measurements, as we do not also have pre-treatment microbiome samples, which would have enabled a longitudinal analysis. Furthermore, these were taken from different locations within plots (see Supp. Results for further discussion). In the upper and lower forest floors, all soil chemistry measurements apart from total N (upper forest floor) and total S and NH^4+^ (lower forest floor) were significantly different (*t*-test *P* ≤ 0.05) between control and treatment samples (Fig. 3; Fig. S3), with pH, total N, and exchangeable (plant available) Ca^2+^, Mg^2+^, Mn^2+^, NH^4+^, and SO^4-^ being higher and organic matter (LOI), total S, total C, Al^3+^, and NO^3-^ levels being lower in treatment than control samples. The magnitude of differences between control and treated sites was typically larger in the upper than the lower forest floor (Fig. S3; Fig. 2; Table S2). In the upper B horizon, only six of the measurements were significantly different between control and treatment samples, with organic matter, total C, total N, and total S being lower and Mn^2+^ and SO^4-^ being higher in treatment than in control samples. Interestingly, the direction of change between treatment and control samples was the same for all significant differences in measurements, aside from total N, where it is significantly increased in lower forest floor treatment samples and significantly decreased in upper B horizon treatment samples. It is also the only measurement that was not significant for the upper forest floor horizon. Taken together, these data demonstrate that the lime treatment effectively increased soil pH, Ca^2+^, Mg^2+^, Mn^2+^, NH^4+^, and SO^4-^ concentrations and decreased total S, Al^3+^, and NO^3-^ concentrations. With regard to organic matter and total C, we observe a decrease in treated soils. Previous studies have observed increases or decreases in total C upon liming (55). The change that we observe is small (4%), which is congruent with other studies that show changes in total C upon liming are observed decades post-treatment, whereas our sampling is after a short time period (56).

### Prokaryotic but not fungal alpha diversity was increased in lime-treated samples

We compared the alpha diversity between samples by grouping them by their treatment (control or treatment) and their soil horizon (upper forest floor, lower forest floor, and upper B horizon) for both the bacterial/archaeal (16S rRNA gene) and fungal data (ITS2 rRNA gene). For bacteria/archaea, there was significantly (ANOVA *P* ≤ 0.05) higher Faith’s phylogenetic diversity in the treatment versus control (Fig. 2A) samples, and in the upper forest floor and upper B horizons in comparison to the lower forest floor (Fig. 3B). When examining differences between control and treatment results in the different horizons, Faith’s phylogenetic diversity was higher in the treatment versus control in both the upper and lower forest floor, but was only significantly so (ANOVA *P* ≤ 0.05) in the upper forest floor, whereas the trend was reversed in the upper B horizon, but statistically significant (Fig. 3C). Similar trends were observed using other alpha diversity metrics: number of ASVs, Chao1 richness, Shannon diversity, and Simpson’s diversity (Fig. S4). In contrast, there were no significant differences between control and treatment samples within the fungal community for any alpha diversity metrics, within any of the soil horizons (Fig. 2D and F; Fig. S5). There was significantly higher (ANOVA *P* ≤ 0.05) richness (number of ASVs and Chao1 richness) in the upper forest floor versus lower forest floor and upper B horizon samples, but alpha diversity indices that also incorporate abundance measurements or evenness showed no differences (Fig. 3E; Fig. S5).

### Shifts in microbial community composition (beta diversity) were observed with lime amendment in prokaryotic but not fungal communities

**Fig 4:**
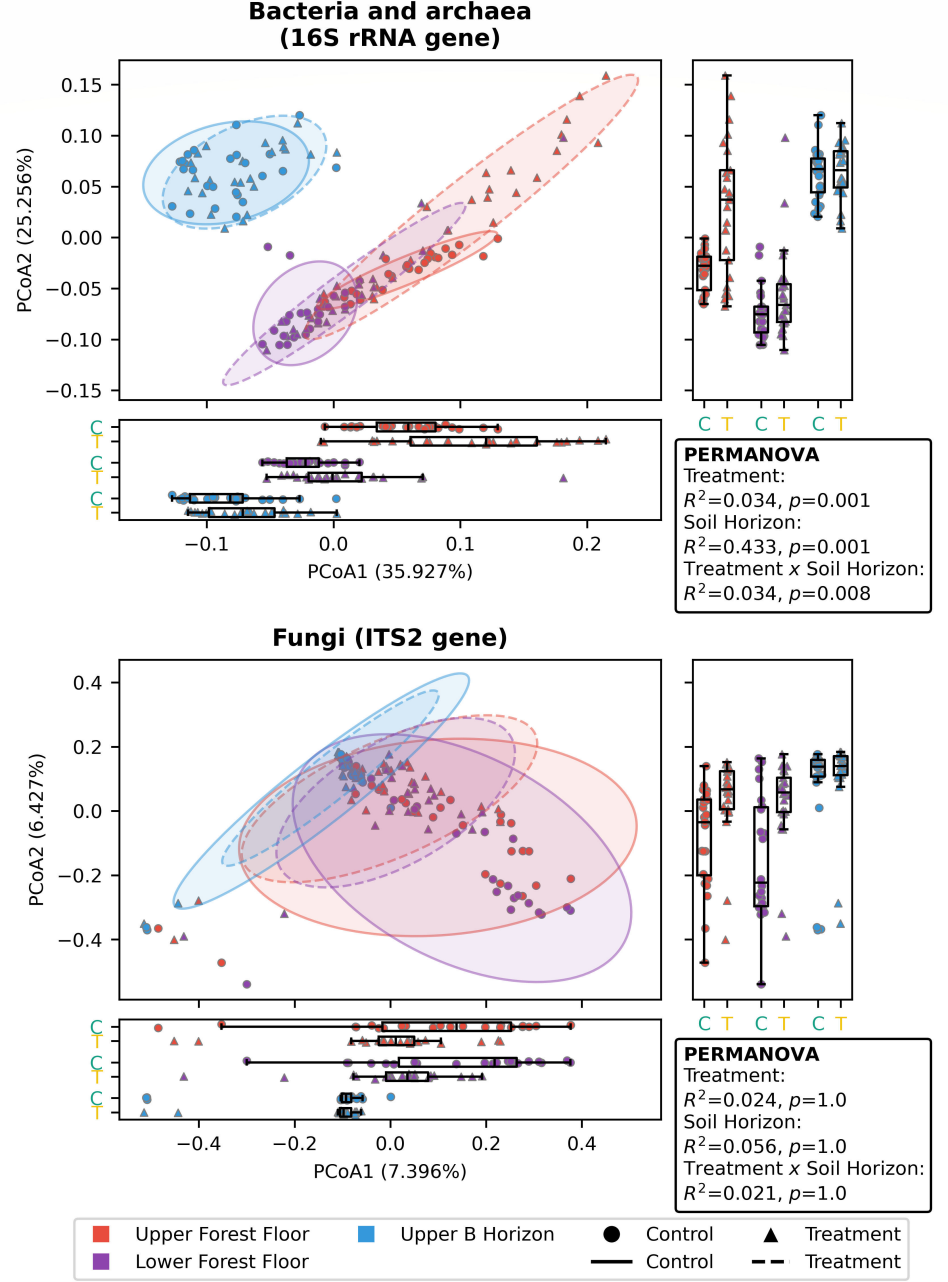
Beta diversity of microbial community samples. PCoA and PERMANOVA tests with beta diversity for bacterial/archaea (top) and fungi (bottom) with all samples grouped by control and treatment within each soil horizon. In each plot, samples are shown as individual points, and ellipses show confidence intervals (two standard deviations) for each group. Values shown on each axis label indicate the proportion of sample variation accounted for by that axis. Box plots along the axes show samples grouped by both soil horizon and treatment. Values shown in the boxes are for PERMANOVA R^2^ and *P*-values for treatment, soil horizon, and the interaction between treatment and soil horizon. Additional beta diversity metrics and all statistical test results are provided in Tables S3 and S4.

We compared the differences in microbial diversity with treatment, as well as between the different soil horizons, using weighted UniFrac distance for the prokaryotic community and Bray-Curtis dissimilarity for the fungal community. PERMANOVA tests included location (control and treatment sites 1–5; Fig. 3), soil horizon (Fig. 1), sample within site (replicate number), and sampling round (July or October) as covariates (Fig. 4; Table S3). Soil chemistry measurements were taken from different replicates within sites and were therefore not included in this analysis. For the bacterial/archaeal community, most of the variation between samples was due to the soil horizon (R^2^ = 0.433, *P* = 0.001, FDR-adjusted *P* = 0.016), although there were also significant differences with lime treatment (R^2^ = 0.034, *P* = 0.001, FDR-adjusted *P* = 0.016) and sampling round (R^2^ = 0.024, *P* = 0.003, FDR-adjusted *P* = 0.031), as well as the number of significant interactions between these variables, including between treatment and soil horizon (R^2^ = 0.034, *P* = 0.008, FDR-adjusted *P* = 0.062; Fig. 4; Table S3). Bray-Curtis dissimilarity was also used for the prokaryotic community, and similar differences were attributed to soil horizon, lime treatment, sampling round, and interactions (Table S3); however, when FDR adjustments were applied, the significance was lost. There is clear clustering of all upper B horizon samples from forest floor samples on both the first (36% variation) and second (25% variation) PCoA axes (Fig. 4), and although there is also clustering of lower forest floor samples away from upper forest floor samples, this difference is not as large. Within the upper forest floor, there is distinct clustering of treatment samples from the control (Fig. 4). By contrast, there is no clear clustering of the treatment from control within either the lower forest floor (Fig. 4) or upper B horizon (Fig. 4). The observed trend of there only being separation of treatment from control within the upper forest floor holds true when looking at each horizon individually (Fig. S6). Beta dispersion analysis was used to determine whether there are significant differences in variation within different soil horizons (betadisp, ANOVA *P* ≤ 0.005) and treatment versus control groups (betadisp, ANOVA *P* ≤ 0.01). However, there was no significant difference in variation within sampling rounds (betadisp, ANOVA *P* > 0.05).

Within the fungal community, there were no significant differences in any of the variables included in the PERMANOVA tests (Fig. 4; Table S4). Unlike in the bacterial/archaeal community, for the fungal community, there was no single variable that accounted for a large proportion of variation between samples (maximum R^2^ = 0.056 for soil horizon). Furthermore, we note that for the fungal ordination plots, only a small amount of variation was shown on the first two axes (7% and 6% for PCoA1 and PCoA2, respectively), indicating that variation between samples is not well represented on two axes. We observed some clustering of upper and lower forest floor samples away from the upper B horizon (Fig. 4), indicating that, as for bacteria/archaea, the upper B horizon is distinct from both forest floor horizons. Additionally, we observed minor clustering of treatment samples from control samples within the upper forest floor and lower forest floor that was not observed in the upper B horizons (Fig. S4). Similarly, the minor clustering of treatment from control is observed when each horizon is examined individually (Fig. S7). We also note that beta dispersion analysis showed that variance within treatment vs control groups was significantly different (betadisp, ANOVA *P* ≤ 0.05), whereas soil horizons and sampling rounds did not have significant differences in variation (betadisp, ANOVA *P* > 0.05).

### Microbial composition of soils from a chronically acidified Nova Scotian coniferous forest

**Fig 5:**
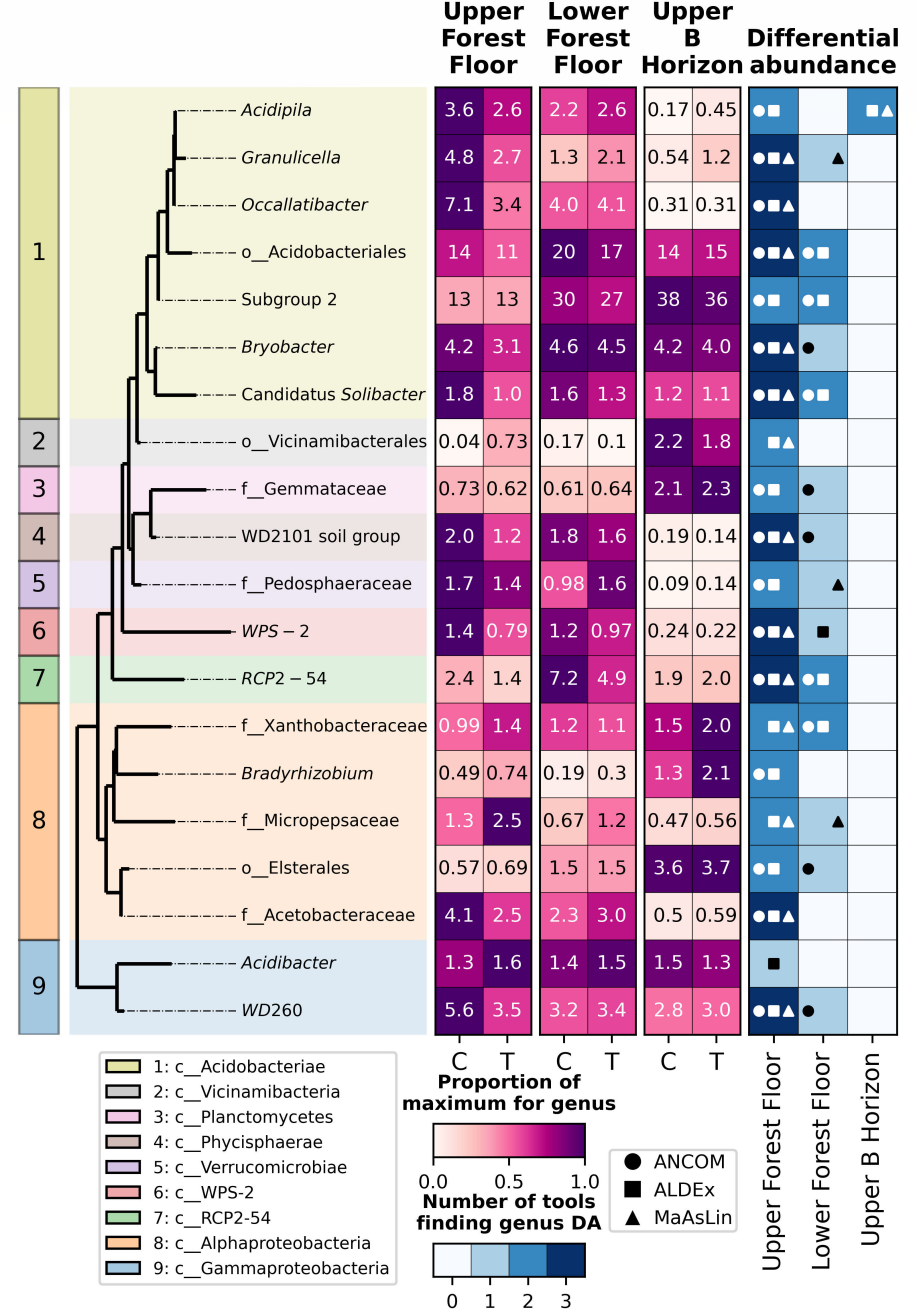
The top 20 most abundant prokaryotic (16S rRNA gene) genera (relative abundance). A phylogenetic tree where genera are colored by the class they belong to (or the lowest taxonomic level classified) (left). Heatmaps (middle) show the relative abundance of genera of interest within each horizon + treatment group. Cell values are the mean value for each soil horizon + treatment group. Results of differential abundance tests (right) show which of the tests (ANCOM, MaAsLin, or ALDEx) found a genus to be significantly differentially abundant (indicated with markers) between control and treatment within each horizon. Boxes are colored by the number of tests that found a significant difference.

**Fig 6:**
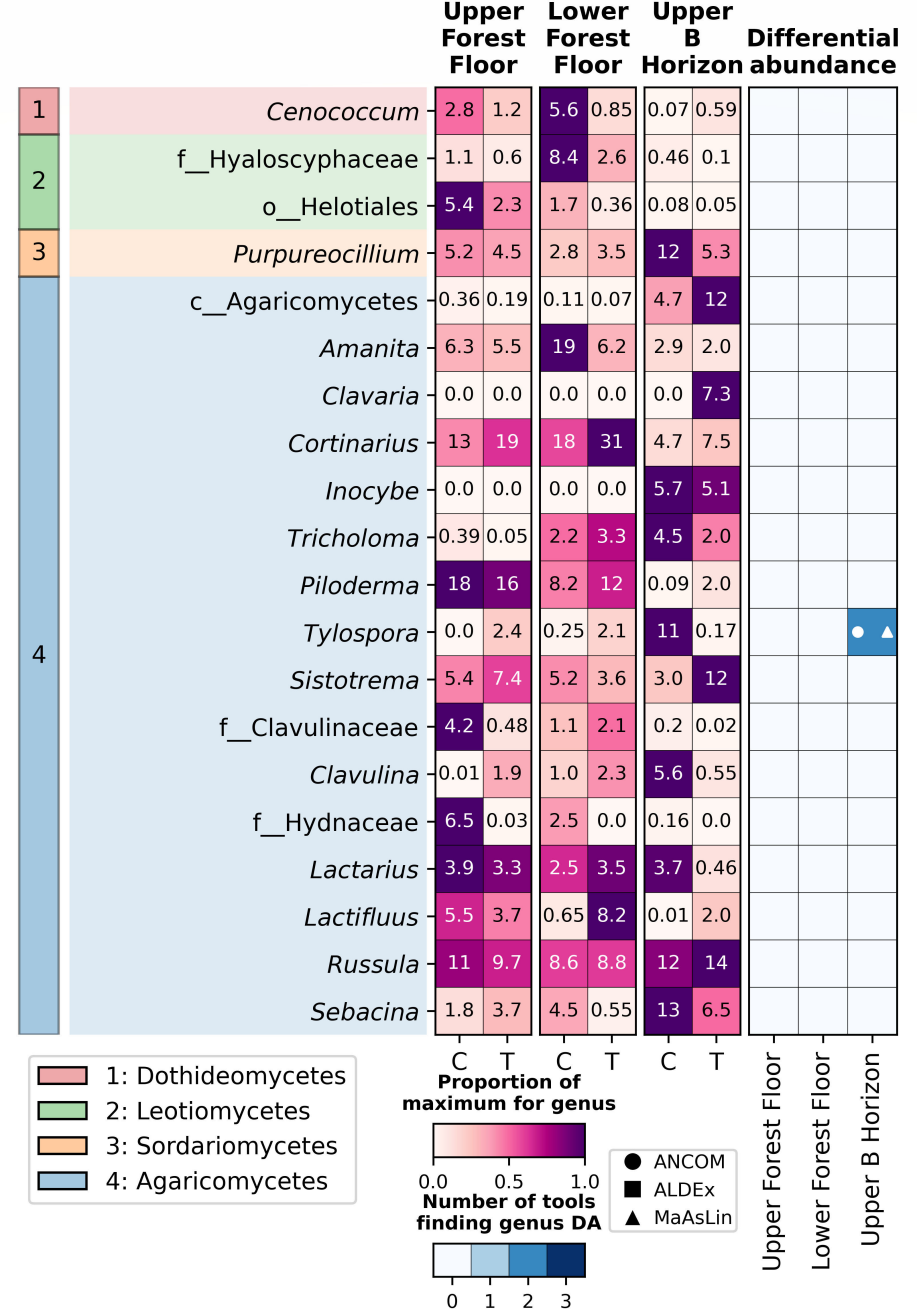
The top 20 most abundant fungal (ITS2 gene) genera (relative abundance). A phylogenetic tree where genera are colored by the class that they belong to (or the lowest taxonomic level classified) (left). Heatmaps (middle) show the relative abundance of genera of interest within each horizon + treatment group. Cell values are the mean value for each soil horizon + treatment group. The results of differential abundance tests (right) show which of the tests (ANCOM, MaAsLin, or ALDEx) found a genus to be significantly differentially abundant (indicated with markers) between control and treatment within each horizon. Boxes are colored by the number of tests that found a significant difference.

We examined the abundance of taxa using both relative abundance, as this is what most studies to date have used, and rCLR abundance, as this accounts for the compositionality of microbiome data (57, 58) (Fig. 5 and 6; Fig. S8 and S9). Tables S5 and S6 show the relative abundance of the 16S/ITS data collapsed at different taxonomic levels across individual samples. In the prokaryotic community, bacteria were dominant, with on average only 0.35% of the community being archaea (from the phyla Crenarchaeota, Nanoarchaeota, and Thermoplasmatota; Table S5). Seven phyla were present in all samples (Proteobacteria, Bacteroidota, Acidobacteriota, Planctomycetota, Verrucomicrobiota, Actinobacteriota, and RCP2-54), and some of these were also high in relative abundance in a large number of samples: (i) Acidobacteriota dominate in all sample types (41%–67% relative abundance); (ii) Proteobacteria are the second most abundant phyla in all sample types (14%–29%); (iii) Bacteroidota are abundant in upper (10%) and lower (3.4%) forest floor treatment but not control (2.9% or 0.99%, respectively) samples; and (iv) Planctomycetota account for ~3.1%–3.9% relative abundance in lower forest floor and upper B horizon samples but are more abundant in upper forest floor samples (5.2%–5.4%; Fig. S8). At lower taxonomic levels, there are many ASVs that were not able to be classified; only 8,114 of the 13,684 16S rRNA gene ASVs had genus-level classifications, with a large number of these being made up of genera that are likely not well characterized, that is, *Candidatus* classifications (431 ASVs) or classifications without Latin scientific names (3,454 ASVs).

The fungal community was dominated by Basidiomycota (78%–90% relative abundance) and by Ascomycota to a lesser extent (8.7%–20%); these were the only two phyla present in all samples (Fig. S9), with all other phyla present at <2% relative abundance on average. Mortierellomycota and Mucoromycota were also highly prevalent (present in 95% of lower forest floor control samples and 100% of all other sample groups, or 60%**-**100% of samples, respectively) but were only present at a maximum relative abundance of 1.7% and 0.89%, respectively. In contrast to the bacterial/archaeal community, where almost all taxa that were abundant were also highly prevalent, in the fungal community, there were very few taxa with genus-level classifications present in >90% of samples within at least one sample grouping; these belonged to the Agaricomycetes (*Galerina*, *Sebacina*, *Amanita*, *Russula,* and *Cortinarius*), Mortierellomycetes (*Mortierella*), and Dothideomycetes (*Cenococcum*). For the fungal ASVs, there was also a large proportion without a genus-level classification (only 1,210 of 1,785 ASVs had a genus-level classification).

### Determining associations between lime treatment and abundant taxa

To identify which genera might be driving differences in community composition shown in ordination plots (Fig. 4), we carried out differential abundance testing between control and treatment samples separately for each soil horizon (Fig. 5 and 6; Tables S7 and S8). Figures S10 and S11 show additional tests that account for the sampling round (Table S9). Because ASVs have a low mean prevalence (16S 4.8% and ITS2 3.7%), we chose to focus on differential abundance testing at the genus level (16S 26.7% and ITS2 14.7% mean prevalence) to draw more meaningful comparisons between treatments. Where taxa were not classified at the genus level, we used the lowest classified taxonomic level. For both the bacterial/archaeal and fungal genera, we carried out differential abundance testing using ALDEx, ANCOM, and MaAsLin, and we consider a genus to be differentially abundant if at least two of these three tests identify it.

### Bacterial/archaeal taxa significantly associated with lime treatment

Overall, 203 genera were identified as being significantly differentially abundant (≥2 tests) between control and treatment samples in at least one of the three horizons assessed; 197 in the upper forest floor, 13 in the lower forest floor, and 8 in the upper B horizon (hereafter, DA refers to significantly differentially abundant with two or more tests) (Tables S7 and S8). When focusing on the top 20 most abundant genera (relative abundance) between control and treatment samples, 19 genera within the upper forest floor, five genera in the lower forest floor, and one genus in the upper B horizon were DA (Fig. 5).

The only genus that was DA in the upper B horizon, *Acidipila* (Acidobacteriae), was slightly more abundant in treatment than control samples, 0.41 vs 0.18%, respectively (Fig. 5). Of the 19 genera identified as being DA in the upper forest floor, 14 were more abundant in the control than treatment samples (Fig. 5). These genera were dispersed across seven different classes with only the Acidobacteriae class showing a consistent trend for all DA genera (more abundant in control than treatment samples) (Fig. 5). In contrast, only five genera (from two classes) identified were more abundant in treatment than in control samples, with four of those being from the Alphaproteobacteria class (Fig. 5). All taxa identified as DA in the lower forest floor were also DA in the upper forest floor. In all but one of these, the direction of change was consistent (i.e., more abundant in control than in treatment samples), but the *Xanthobacteraceae* family was slightly more abundant in treatment than in control samples in the upper forest floor (1.5 versus 0.89%, respectively), although they were slightly more abundant in control than in treatment samples in the lower forest floor (means of 1.16% in both, but medians of 0.92 and 0.16%, respectively).

We also carried out differential abundance testing that accounted for the two different sampling rounds (October and July). All taxa that were DA when considering only treatment versus control within each horizon were no longer DA; however, there were some significant interactions between treatment and sampling round for three genera (Fig. S10): *Bryobacter*, Candidatus *Solibacter* (both Acidobacteriae), and *WD260* (Gammaproteobacteria), all of which were more abundant in control than treatment samples. No genera were DA with sampling round alone (Table S9).

### Fungal taxa significantly associated with lime treatment

In total, nine fungal genera were identified as significantly differentially abundant by two or more DA tests in control versus treatment samples across all horizons (Tables S7 and S8). Of those genera, seven were identified within the upper forest floor, one within the upper B horizon, and one in both the upper and lower forest floor. However, eight of these genera have very low relative abundance; hence, although there are differences between control and treatment samples, they are quite small, calling into question the biological significance of this change for the overall fungal community (Table S8). The only significantly DA genus within the top 20 most abundant taxa was *Tylospora*, which was found to be significantly differentially abundant in the upper B horizon and was more abundant in control than in treatment samples (Tables S7 and S8; Fig. 6). Similarly, when sampling round is considered, *Tylospora* was identified by the same two tests (Fig. S11; Table S9). No test found any significant differences in genera when looking at the interaction of treatment and sampling rounds.

## DISCUSSION

Through the measurement of soil chemistry data and amplicon sequencing data for prokaryotes and fungi, we have evaluated the impact of lime treatment within an acidified Nova Scotia coniferous forest. We observed large shifts (alpha and beta diversity) in the prokaryotic community between each of the soil horizons assessed, with the largest differences between control and treatment samples being observed in the upper forest floor horizon (Fig. 3 to 5). In contrast to the prokaryotic community, there were very few differences found in the fungal community between either control and treatment samples or between different soil horizons (Fig. 2, 4, and 6). Our results are similar to two earlier studies where coniferous forests were treated with liming amendments, despite differences in study design. Cha et al. (20) applied lime with dosages calculated by local pH, organic matter, and soil texture to five forest sites in Korea (one *Pinus koraiensis* and four *Pinus densiflora*), whereas Cruz-Paredes et al. (21) applied a wide gradient of wood ash to a *Picea abies* plantation in Denmark. Microbial sampling was carried out 4–8 years or 1-year post-treatment, respectively. Both studies observed minimal or no changes in fungal soil communities and larger changes in prokaryotic/bacterial communities with treatment (20, 21). We note that these data are in contrast to the findings of Sridhar et al*.* (22), who found large shifts in fungal communities upon liming. Although these differences are puzzling, they may reflect the differences in the choice of ITS primers that were used in these studies. We have used the ITS2 primers—as did the study by Cruz-Paredes et al. (21)—Sridhar and coworkers (22) used ITS1, whereas Cha and co-workers (20) used ITS3 and ITS4. It is possible that these differences reflect the inherent limitations in ITS sequencing (59).

Higher prokaryotic alpha diversity, as observed in this study between treatment and control upper (*P* ≤ 0.005) and lower forest floor samples (not statistically significant; Fig. 2), has been associated with lime treatments in a previous study (20), as well as with healthier plants (60). We also found significant shifts in prokaryotic community composition (beta diversity; Fig. 4) that were associated with treatment, soil horizon, and sampling round, but not with location (sites 1-5), despite variation in between-site lime application rates noted earlier.

Curiously, we found no significant interaction between treatment and location (Table S2), whereas Cha et al. (20) found inconsistent differences in microbial alpha diversity and composition between controls and lime-treated sites and associated these differences with variation in soil chemistry measurements between the sites. This may be due to their sites being treated with different amounts of lime and sampled at different post-treatment times (range: 4–8 years). Consistent with other studies (20, 21), no variable was found in this study to be significantly associated with fungal community structure (Fig. 4; Table S3).

We found that several genera within the Acidobacteriae class (Acidobacteriota phylum, previously Acidobacteria) were significantly less abundant in treatment than in control samples, whereas several genera within the Alphaproteobacteria class (Pseudomonadota phylum, previously Proteobacteria) were significantly more abundant in treatment versus control samples (Fig. 5). Cruz-Paredes et al. also identified the same trend in Acidobacteria, reporting that increasing wood ash treatment led to decreases in Acidobacteria (21). Cha et al. identified a similar trend in Proteobacteria, showing that with increases in pH associated with liming, there was also an increase in Proteobacteria (20).

Acidobacteriae, as the name suggests, are known to be associated with acidic soils and are capable of utilizing a variety of carbon sources and nitrogen metabolism mechanisms (4, 61, 62). Consistent with this, we found several Acidobacteriae taxa decreased in relative abundance with liming, along with associated increases in pH. Although the pH did increase (Fig. 2), it is still acidic, which is likely the reason that Acidobacteriae remain abundant in the treatment sites despite a significant decrease in their relative abundance (Fig. 5). Alphaproteobacteria, a dominant class of soil bacteria, are reported to respond variably to pH changes, with some groups reporting a stronger association with lower pH (63) or acid treatment (64) and others with increased pH (65, 66). In agreement with related literature (63–66), we find that Alphaproteobacteria are more abundant in treatment (pH 4, Table S1; Fig. 5) than control sites (average pH 2.5, Table S1; Fig. 5), suggesting that although Alphaproteobacteria are still present at mildly acidic pHs, they tend to increase in abundance with increasing pH.

*Tylospora* was the only abundant fungal genus that we identified by any differential abundance test, with it being less abundant in treated than in control samples in the upper B horizon (0.15% and 9.3%, respectively). Cruz-Paredes et al. also identified *Tylospora* as decreasing in relative abundance in their wood-ash-treated samples (21), and Kjøller et al. also reported in their meta-analysis (primarily consisting of studies focused on Nordic forests) that within *Tylospora*, a genus of ectomycorrhizal fungi, differences were species-specific, with some decreasing with both lime and wood ash treatment, whereas others remained consistent (67).

Tools, such as PICRUSt2 (68), are often used to predict functional potential within bacterial communities. However, these tools need reasonably close matches at the ASV level for predictions to be valid. Currently, many environmental bacteria have not been formally classified or studied, potentially resulting in poor resolution of taxonomic classifications (69). Many of the bacteria identified in our study are from taxa without genus or even family level classifications. As a result, efforts to perform functional predictions are premature, and we were unable to predict functional capacity within these microbiomes.

We acknowledge and identify limitations in our study that, if addressed, should improve similar future studies. A helicopter is an imperfect method of applying crushed limestone, as it is impossible to ensure uniform distribution across a wooded area. Figure 2 demonstrates some unevenness in soil chemistry changes upon treatment; these differences may reflect differences in lime dosage. Microbiome studies using sequencing data are inherently compositional, and because of this, we exclusively use relative abundance or centered log ratios as opposed to absolute abundance (57, 58). Microbiome sampling started at our study site after liming had occurred and, as such, precluded any pre-treatment sampling and analysis from being completed. The single round of post-treatment soil chemistry sampling (October 2019) was also done prior to the start of microbiome sampling; although these soil samples were collected within the same plots and soil horizons as microbiome samples, this difference in timing prevented soil chemistry data from being fully integrated into our analysis. Also, as discussed above, two microbiome sampling rounds were conducted (October and July), and the sampling round was found to have a small but significant impact on prokaryotic community composition (Weighted UniFrac PERMANOVA R^2^ = 0.024, *P* = 0.003). However, genus-level differential abundance testing for control versus treatment lost significance when the sampling round was included. Sampling intensity was relatively low in each sampling round, which, if increased, may allow for more genera to be identified as DA. We predict that in future years, there will be further shifts in the upper forest floor, and as the crushed limestone works its way down to the lower forest floor and upper B horizon depths, there will also be shifts in these bacterial/archaeal soil communities. Consistent with this notion, Sridhar et al. (22) found that fungal taxa became more differentially abundant over time (2 years versus 25 years post-liming treatment).

Our results suggest that changes in the bacterial/archaeal communities of the upper forest floor may serve as an early indicator of recovering soil health in response to liming. Studies that further develop this research can better determine whether these shifts in microbial composition are indeed both long-term and beneficial. Additionally, future studies should aim to assess microbial community changes over longer timescales, both pre- and post-liming, and better integrate soil chemistry measurements for every microbial community characterization. This could enable identification of driving factors for positive changes in microbial communities that could then be tied to more easily measured soil health indicators. Furthermore, incorporating meta-genomic, -transcriptomic, and -proteomic studies will help elucidate changes that are not observed by taxonomic changes alone, particularly when taxonomic annotation is lacking in environmental studies.

## Data Availability

All raw sequencing data have been deposited under the European Nucleotide Archive BioProject PRJEB58425. All code used for data analysis, performing statistical tests, and generating figures is on Github (https://github.com/M-Hosmer/NS_LimingTrial_OtterPond).

## SUPPLEMENTAL MATERIAL

Table S1 aem.02171-24-s0001.xlsx Soil chemistry.

Supplemental material aem.02171-24-s0002.docx Supplemental results, Table S2, and Fig. S1 to S11.

Tables S3 and S4 aem.02171-24-s0003.xlsx PERMANOVA statistical tests for the 16S dataset.

Table S5 aem.02171-24-s0004.xlsb 16S relative abundance at all taxonomic levels.

Table S6 aem.02171-24-s0005.xlsx ITS2 relative abundance at all taxonomic levels.

Table S7 aem.02171-24-s0006.xlsx Differential abundance test for control vs. treatment within the upper B horizon for the ITS2 data.

Table S8 aem.02171-24-s0007.xlsx Differentially abundant ITS taxa.

Table S9 aem.02171-24-s0008.xlsx Differential abundance test for time points within the upper B horizon for the ITS2 data.

ASM does not own the copyrights to Supplemental Material that may be linked to, or accessed through, an article. The authors have granted ASM a non-exclusive, world-wide license to publish the Supplemental Material files. Please contact the corresponding author directly for reuse.
