# Supplementary material for: Lime amendment to chronically acidified forest soils results in shifts in prokaryotic communities
Source: Appl Environ Microbiol. 2025 Dec 29;92(1):e02171-24. doi: 10.1128/aem.02171-24 (PMC12838336; doi:10.1128/aem.02171-24)
Supplement: Supplemental material — Supplemental results, Table S2, and Fig. S1 to S11. [file aem.02171-24-s0002.docx]

**Supplementary Results: Effect of liming treatment on soil chemistry**

Soil chemistry measurements were taken both pre- and post-treatment. We observe a significant difference between treatment samples pre- and post-treatment (but not control samples) for pH (upper forest floor), organic matter (upper forest floor), total C (upper forest floor), total S (lower forest floor), NO^3-^ (upper B horizon), Ca^2+^ (upper forest floor), Mg^2+^ (upper and lower forest floor), Al^3+^ (lower forest floor) and Mn^2+^ (upper and lower forest floor). We observe a difference between control samples pre- and post-treatment (but not treatment samples) for organic matter (lower forest floor), total C (lower forest floor), total N (upper forest floor), total S (upper B horizon), NH^4+^ (upper B horizon) and SO^4-^ (upper forest floor and upper B horizon). We observe differences between pre- and post-treatment for both control and treatment samples for NO^3-^ (upper and lower forest floor), NH^4+^ (lower forest floor), SO^4-^ (lower forest floor) and Ca^2+^ (lower forest floor)(Supp. Figure 1).

We calculated the percentage change in the measurements between pre- and post-treatment and compared whether the change was larger for the treatment or control samples (Supp. Figure 2). Only SO^4-^ showed significant differences for all three soil horizons (control samples decreased more post-treatment than treatment samples) while total N, NO^3-^ and Al^3+^ were not significant for any soil horizons. The remaining measurements were all significant for only one soil horizon, with total C and organic matter decreasing and Ca^2+^, Mn^2+^ and Mg^2+^ increasing for treatment *vs* control samples in the upper forest floor, total S decreased and pH increased for treatment *vs* control samples in the lower forest floor, and NH^4+^ increased for treatment *vs* control samples in the upper B horizon.

We are hesitant to over interpret the soil chemistry findings and their impact on the microbiome analysis for several reasons: one, there was only a single replicate taken pre-treatment from each of the sites for soil chemistry and no microbiome sampling done. Second, we have no sampling round where both soil chemistry and microbiome samples taken. Finally, while the soil chemistry sampling and microbiome sampling were completed at the same plots, the location within plots was not constant between sampling rounds. The soil chemistry data represents a direct impact from the liming whereas the microbiome is indirect.

**Supplementary Table 2:** Post-treatment soil chemistry measurements and T-test statistics for control and treatment samples within all three horizons.

| **Measure** | **Upper Forest Floor** | | | **Lower Forest Floor** | | | **Upper B Horizon** | | |
| --- | --- | --- | --- | --- | --- | --- | --- | --- | --- |
|  | ***Control*** | ***Treatment*** | ***T-test*** | ***Control*** | ***Treatment*** | ***T-test*** | ***Control*** | ***Treatment*** | ***T-test*** |
| **pH** | 2.4653 (0.1053) | 3.958 (0.5503) | T=-9.97, p<0.0001 | 2.3627 (0.0603) | 2.7487 (0.3386) | T=-4.2, p=0.0002 | 3.5967 (0.1374) | 3.5647 (0.1815) | T=0.53, p=0.6031 |
| **Organic matter (%)** | 98.5467 (0.2446) | 94.7533 (1.8355) | T=7.66, p<0.0001 | 97.8333 (0.9755) | 95.3933 (1.1722) | T=5.99, p<0.0001 | 13.9927 (3.7061) | 11.4787 (2.144) | T=2.2, p=0.0365 |
| **Total C (%)** | 52.62 (0.3833) | 50.34 (1.2322) | T=6.61, p<0.0001 | 52.24 (0.7973) | 51.06 (0.8724) | T=3.74, p=0.0008 | 5.3247 (1.4048) | 4.2067 (0.9398) | T=2.47, p=0.0196 |
| **Total N (%)** | 1.0497 (0.1377) | 1.12 (0.1137) | T=-1.47, p=0.152 | 1.1839 (0.1686) | 1.324 (0.1302) | T=-2.46, p=0.0203 | 0.3299 (0.1875) | 0.1726 (0.0487) | T=3.04, p=0.0051 |
| **Total S (%)** | 0.1629 (0.0253) | 0.1411 (0.028) | T=2.16, p=0.0393 | 0.2073 (0.0393) | 0.1993 (0.026) | T=0.63, p=0.534 | 0.0429 (0.0145) | 0.0291 (0.0092) | T=2.99, p=0.0058 |
| **Ca^2+^ (cmol kg^-1^)** | 0.3055 (0.1584) | 1.6491 (0.3995) | T=-11.7, p<0.0001 | 0.1249 (0.1125) | 0.7133 (0.3791) | T=-5.57, p<0.0001 | 0.0076 (0.0052) | 0.0493 (0.0961) | T=-1.62, p=0.1159 |
| **Mg^2+^ (cmol kg^-1^)** | 0.4985 (0.0747) | 3.0579 (2.5823) | T=-3.71, p=0.0009 | 0.5614 (0.132) | 1.4389 (0.4323) | T=-7.26, p<0.0001 | 0.0125 (0.0028) | 0.0565 (0.0895) | T=-1.84, p=0.0771 |
| **Al^3+^ (cmol kg^-1^)** | 0.3713 (0.2343) | 0.1593 (0.1096) | T=3.07, p=0.0048 | 0.8406 (0.5923) | 0.4794 (0.1472) | T=2.21, p=0.0351 | 2.9299 (0.5684) | 3.2638 (0.6155) | T=-1.49, p=0.1471 |
| **Mn^2+^ (cmol kg^-1^)** | 0.0251 (0.0109) | 0.0698 (0.0217) | T=-6.88, p<0.0001 | 0.0105 (0.0057) | 0.0237 (0.0115) | T=-3.81, p=0.0007 | 0.0014 (0.001) | 0.0041 (0.0041) | T=-2.45, p=0.0208 |
| **NO^3-^ (cmol kg^-1^)** | 0.0002 (0.0001) | 0.0001 (0.0) | T=6.24, p<0.0001 | 0.0002 (0.0001) | 0.0001 (0.0001) | T=5.89, p<0.0001 | 0.0001 (0.0001) | 0.0 (0.0) | T=1.72, p=0.0963 |
| **NH^4+^ (cmol kg^-1^)** | 0.0292 (0.0144) | 0.044 (0.0195) | T=-2.28, p=0.0303 | 0.0203 (0.0062) | 0.0218 (0.0055) | T=-0.65, p=0.5205 | 0.0063 (0.0018) | 0.005 (0.0018) | T=1.98, p=0.0578 |
| **SO^4-^ (cmol kg^-1^)** | 0.0321 (0.0073) | 0.0392 (0.0054) | T=-2.95, p=0.0063 | 0.0284 (0.0061) | 0.0359 (0.0051) | T=-3.52, p=0.0015 | 0.027 (0.0053) | 0.0334 (0.0069) | T=-2.77, p=0.0099 |

Values within Control and Treatment columns show means (standard deviation) across three biological replicates for each of five sites. All replicates are plotted in Supp. Figure 3. Green shading indicates a significant (*p* ≤ 0.05) unpaired t-tests between control and treatment samples.

| 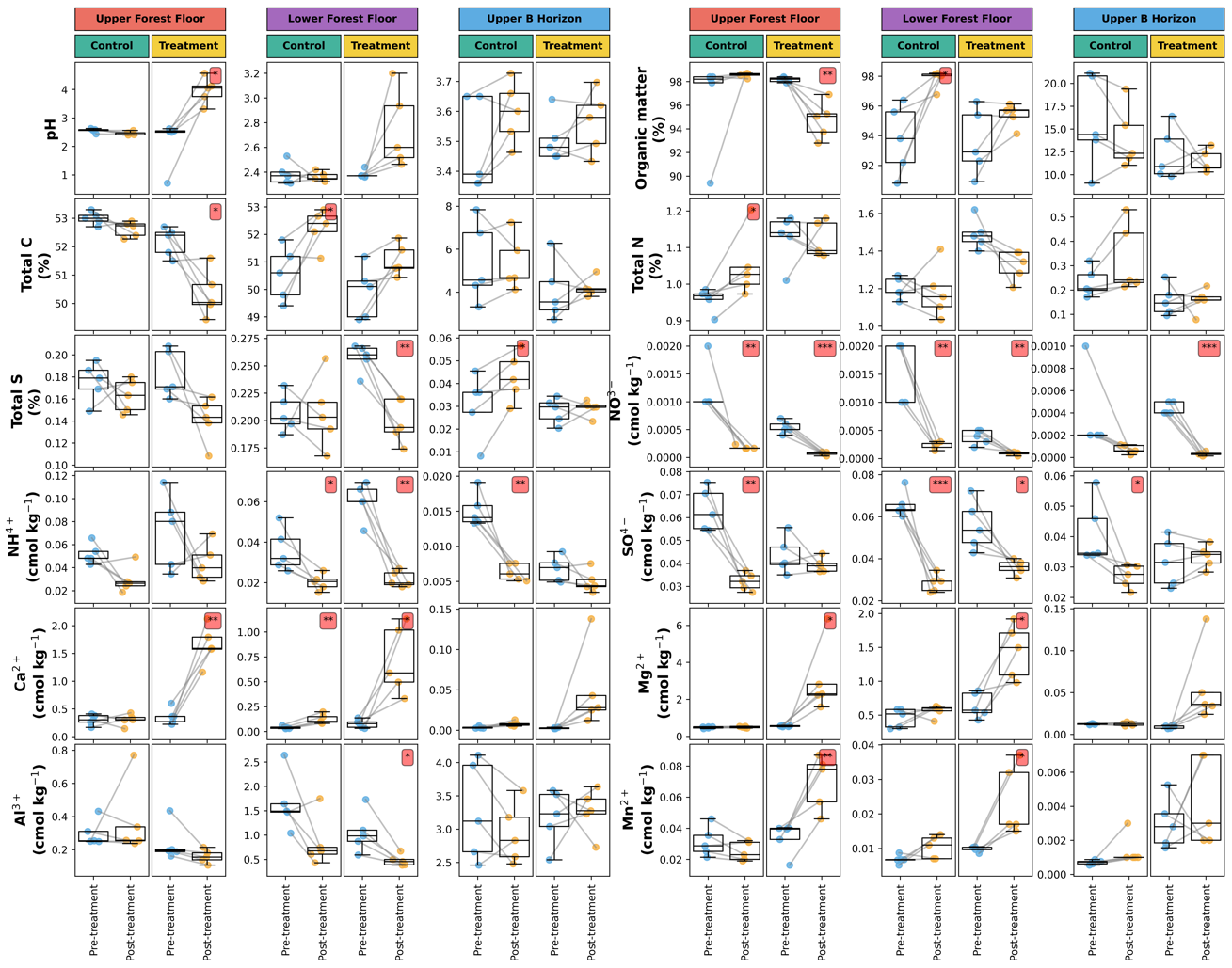  **Supp. Figure 1.** Soil chemistry measurements taken pre- and post-treatment with crushed lime. Plots are separated for control and treatment samples within each of the three soil horizons. Points shown are for a single measurement or the mean of three measurements and lines link measurements taken from the same site. Boxplots indicate the median, upper and lower quartiles. Significant differences between pre-treatment and post-treatment (determined with paired t-tests) are shown by red boxes with * indicating *p* $\leq$ 0.05, ** indicating *p* $\leq$ 0.01 and *** indicating *p* $\leq$ 0.001. |
| --- |

| 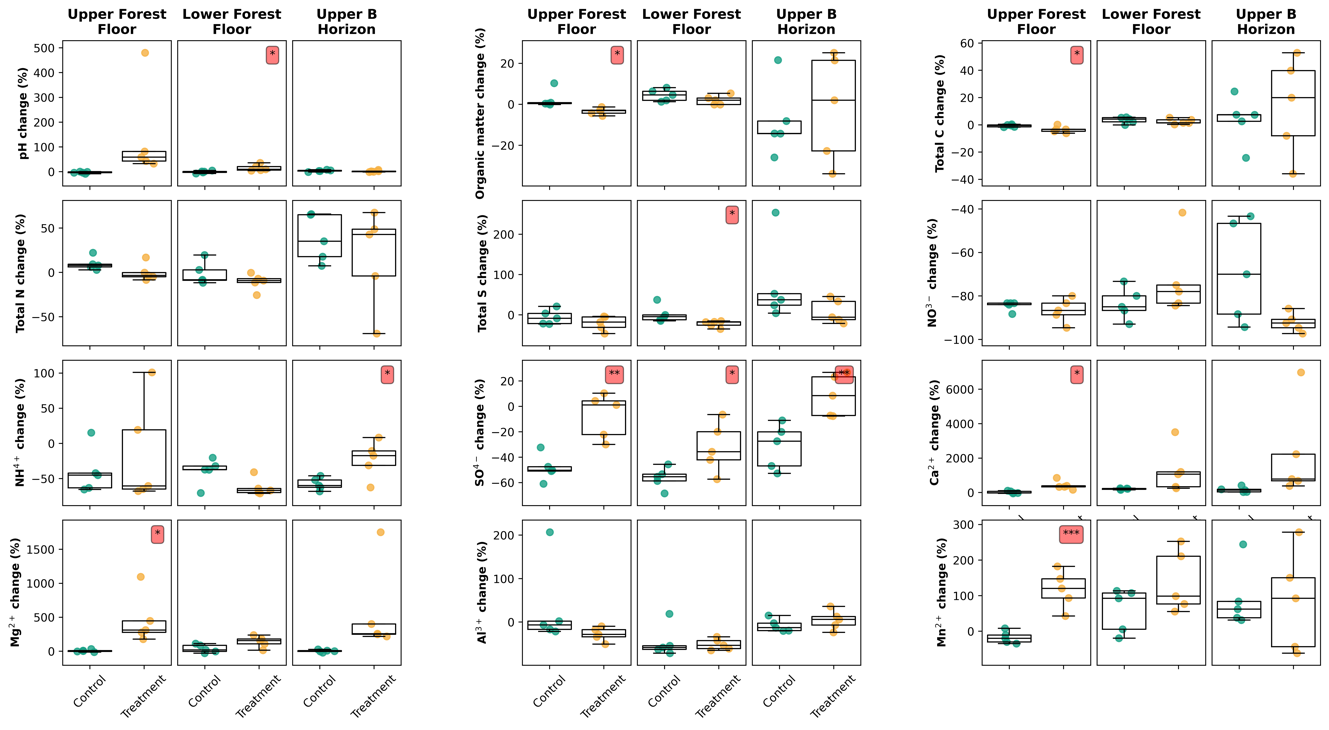  **Supp. Figure 2.** Differences between soil chemistry measurements taken pre- and post-treatment with crushed lime. Points shown represent the percent difference between pre- and post-treatment samples with positive percentages indicating that there was an increase between after liming treatment and negative percentages indicating a decrease after liming treatment. Differences are calculated for each site using the single measurement for pre-treatment samples and the mean of three measurements for post-treatment samples. Boxplots indicate the median, upper and lower quartiles. Significant differences between control and treatment samples (determined with unpaired t-tests) are shown by red boxes with * indicating *p* $\leq$ 0.05, ** indicating *p* $\leq$ 0.01 and *** indicating *p* $\leq$ 0.001. |
| --- |

| 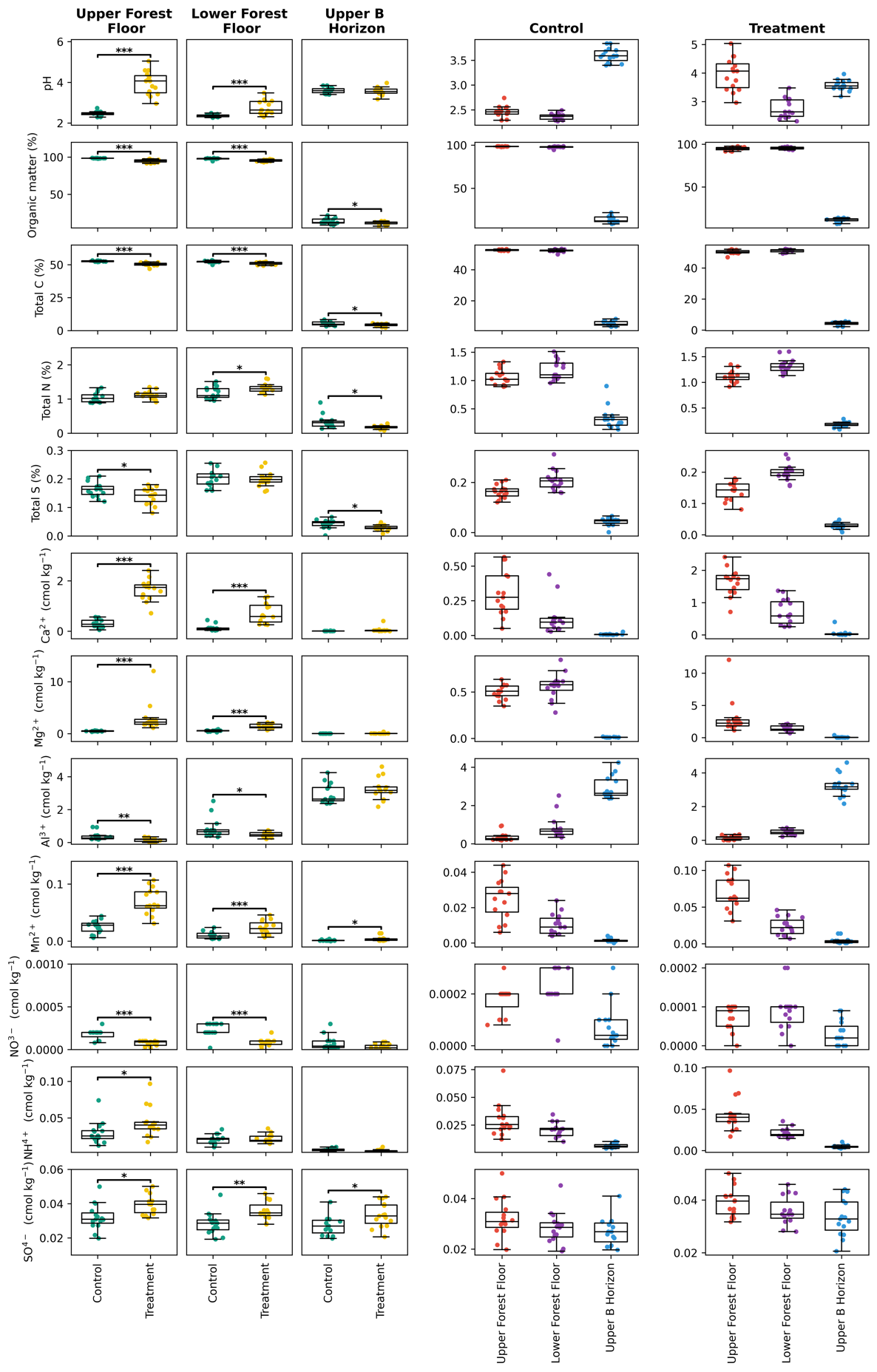  **Supp. Figure 3.** Measurements of pH, LOI (organic matter), total C, total N, total S, Ca^2+^, Mg^2+^, Al^3+^, Mn^2+^, NO^3-^, NH^4+^ and SO^4-^ in control and treatment Upper Forest Floor, Lower Forest Floor and Upper B Horizon samples. Points shown are for three biological replicates at five different sites, and boxplots indicate the median, upper and lower quartiles. Significant differences between control and treatment samples were determined with unpaired t-tests, with * indicating *p* $\leq$ 0.05, *p* $\leq$ 0.005 and *p* $\leq$ 0.001. Unpaired T-tests presented in Supp. Table 2. |
| --- |

| 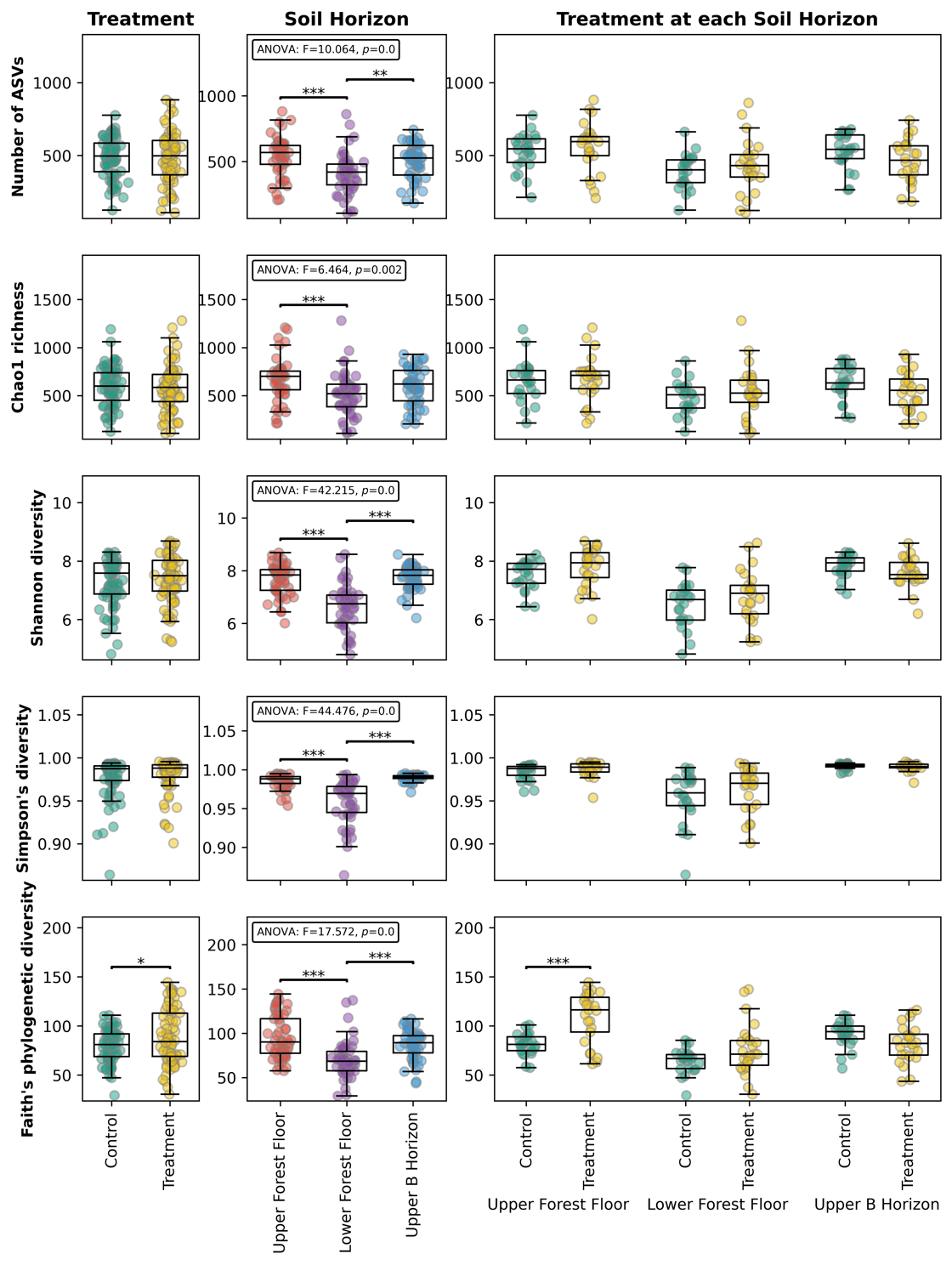  **Supp. Figure 4: Prokaryotic alpha diversity.**  Box plots show alpha diversity metrics at the ASV level with samples grouped to treatment (left), soil horizon (middle) or treatment within soil horizon (right). From top to bottom, the number of ASVs, Chao1 richness, Shannon diversity, Simpson’s diversity and Faith’s phylogenetic diversity are shown. In each of these plots, each sample is shown as an individual point and boxes show the median, upper and lower quartiles while whiskers show the range of the data (1.5 times the interquartile range). Significant differences between treatments (ANOVA test) are denoted by asterisks, with * denoting that *p* ≤ 0.05, ** that *p* ≤ 0.01 and *** that *p* ≤ 0.005. |
| --- |

| 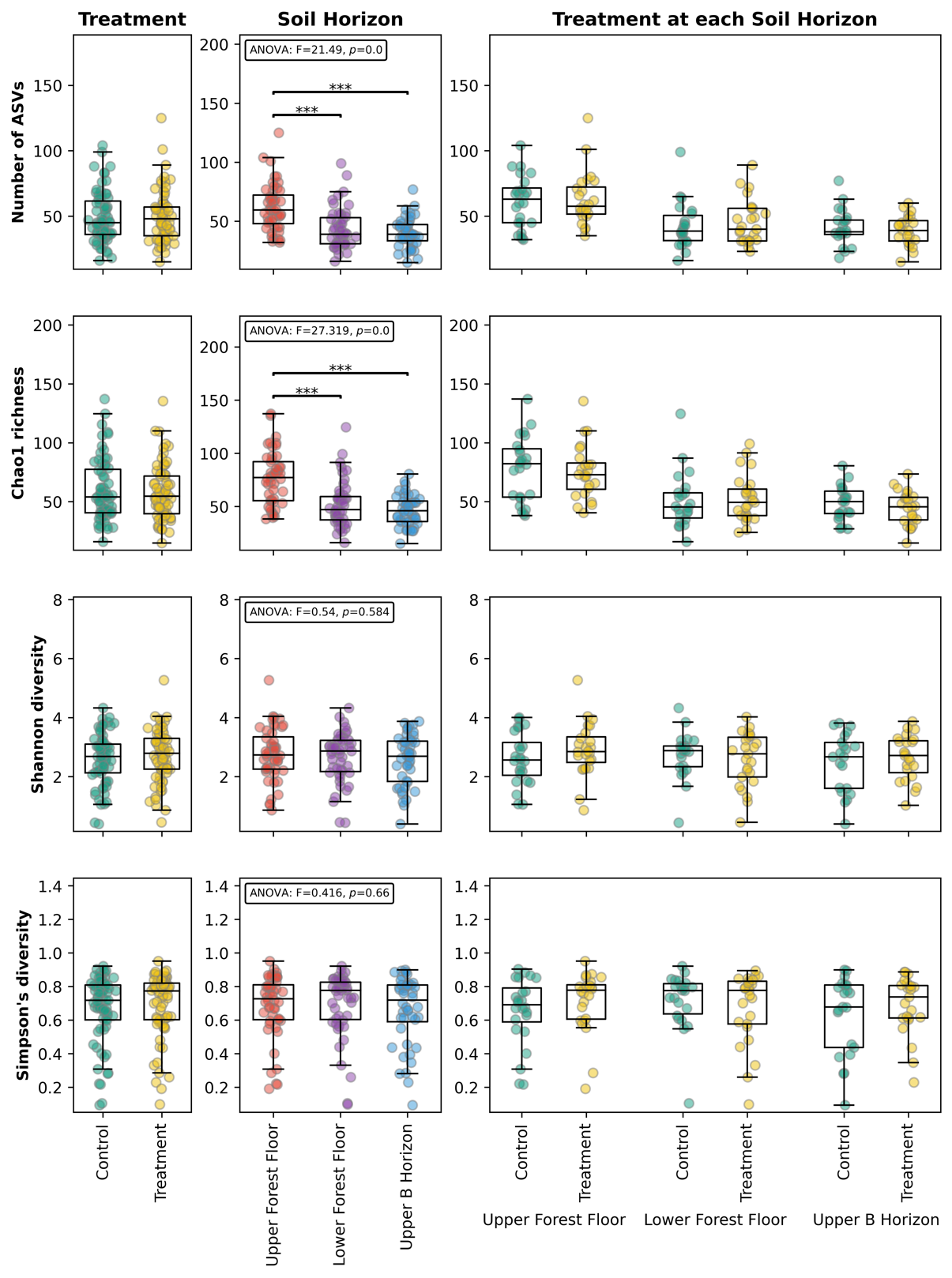  **Supp. Figure 5: Fungal alpha diversity.**  Box plots show alpha diversity metrics at the ASV level with samples grouped to treatment (left), soil horizon (middle) or treatment within soil horizon (right). From top to bottom, the number of ASVs, Chao1 richness, Shannon diversity, and Simpson’s diversity are shown. In each of these plots, each sample is shown as an individual point and boxes show the median, upper and lower quartiles while whiskers show the range of the data (1.5 times the interquartile range). Significant differences between treatments (ANOVA test) are denoted by asterisks, with * denoting that *p* ≤ 0.05, ** that *p* ≤ 0.01 and *** that *p* ≤£ 0.005.   \| 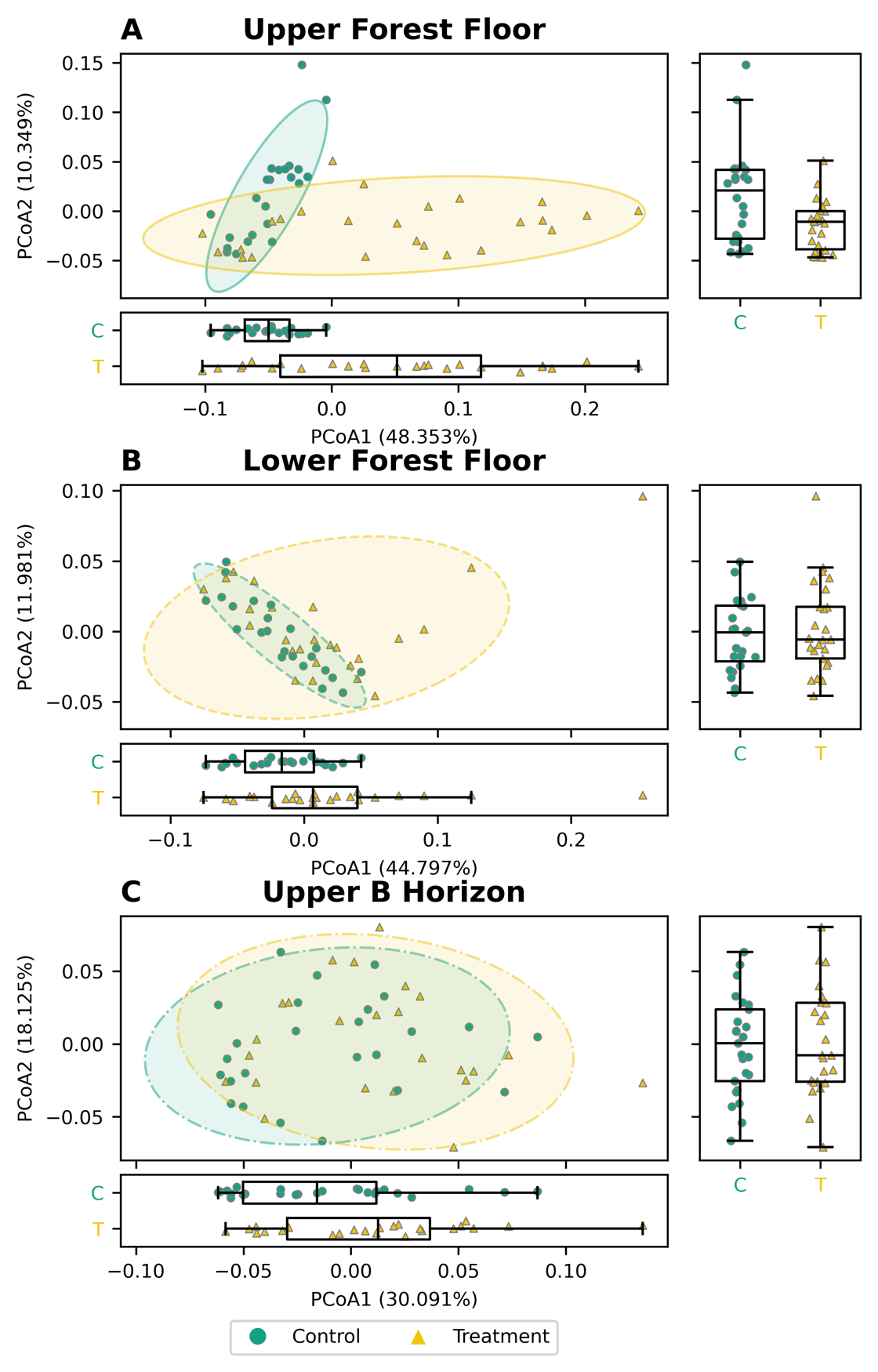 \| \| --- \| \| **Supp. Figure 6:** **Beta diversity of bacterial/archaeal samples divided by horizon.** Principal Coordinates Analysis (PCoA) with beta diversity for bacterial/archaea. Individual plots for each horizon shown with all samples grouped by control and treatment. In each plot, samples are shown as an individual points and ellipses show confidence intervals (2 standard deviations) for each group. Values shown on each axis label indicate the proportion of sample variation accounted for by that axis. Box plots along the axes show samples grouped by both soil horizon and treatment. \|  \| 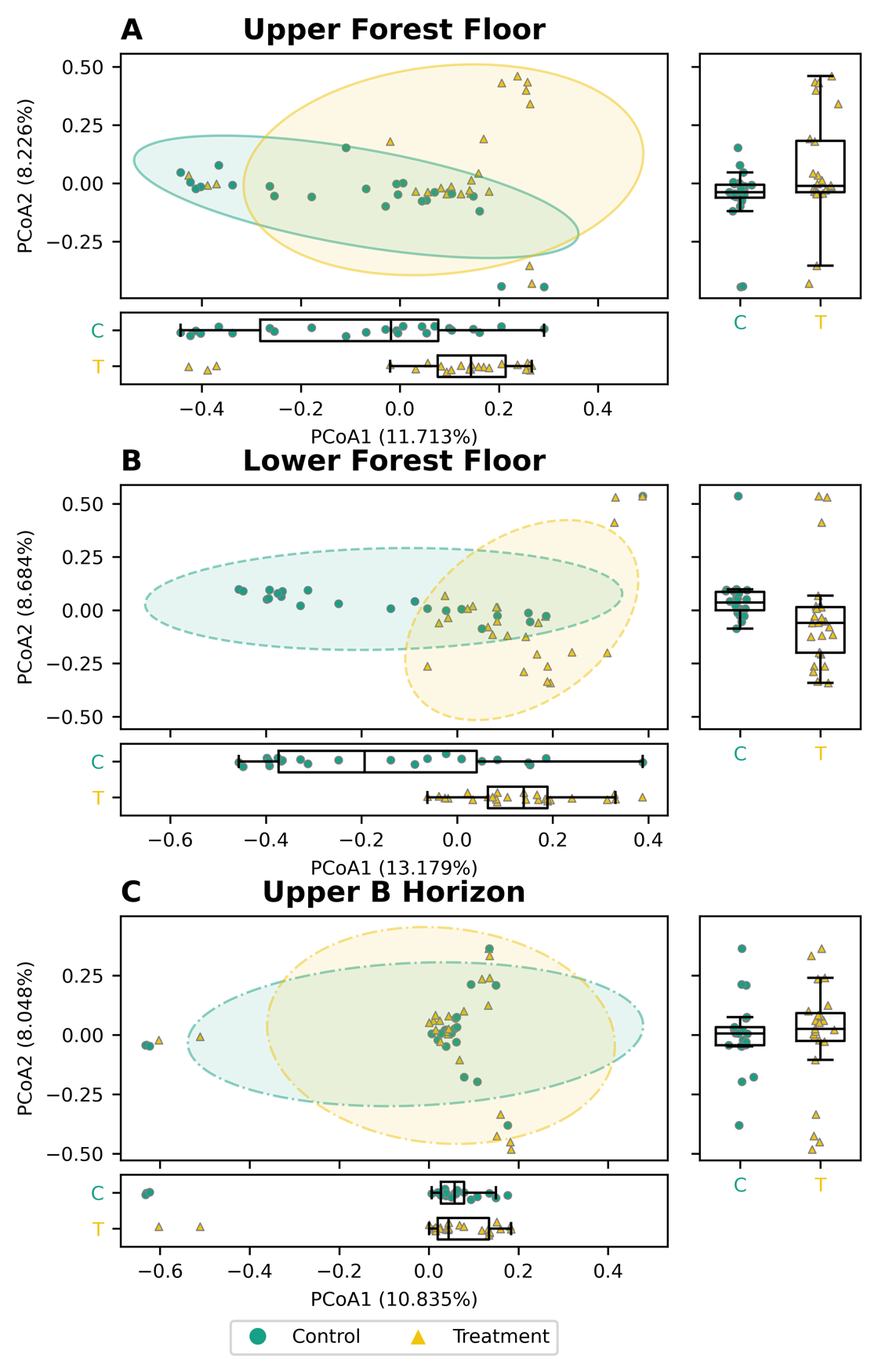 \| \| --- \| \| **Supp. Figure 7: Beta diversity of fungal samples divided by horizon.** Principal Coordinates Analysis (PCoA) with beta diversity for fungi. Individual plots for each horizon shown with all samples grouped by control and treatment. In each plot, samples are shown as an individual points and ellipses show confidence intervals (2 standard deviations) for each group. Values shown on each axis label indicate the proportion of sample variation accounted for by that axis. Box plots along the axes show samples grouped by both soil horizon and treatment. \| |
| --- | --- | --- | --- | --- |

| **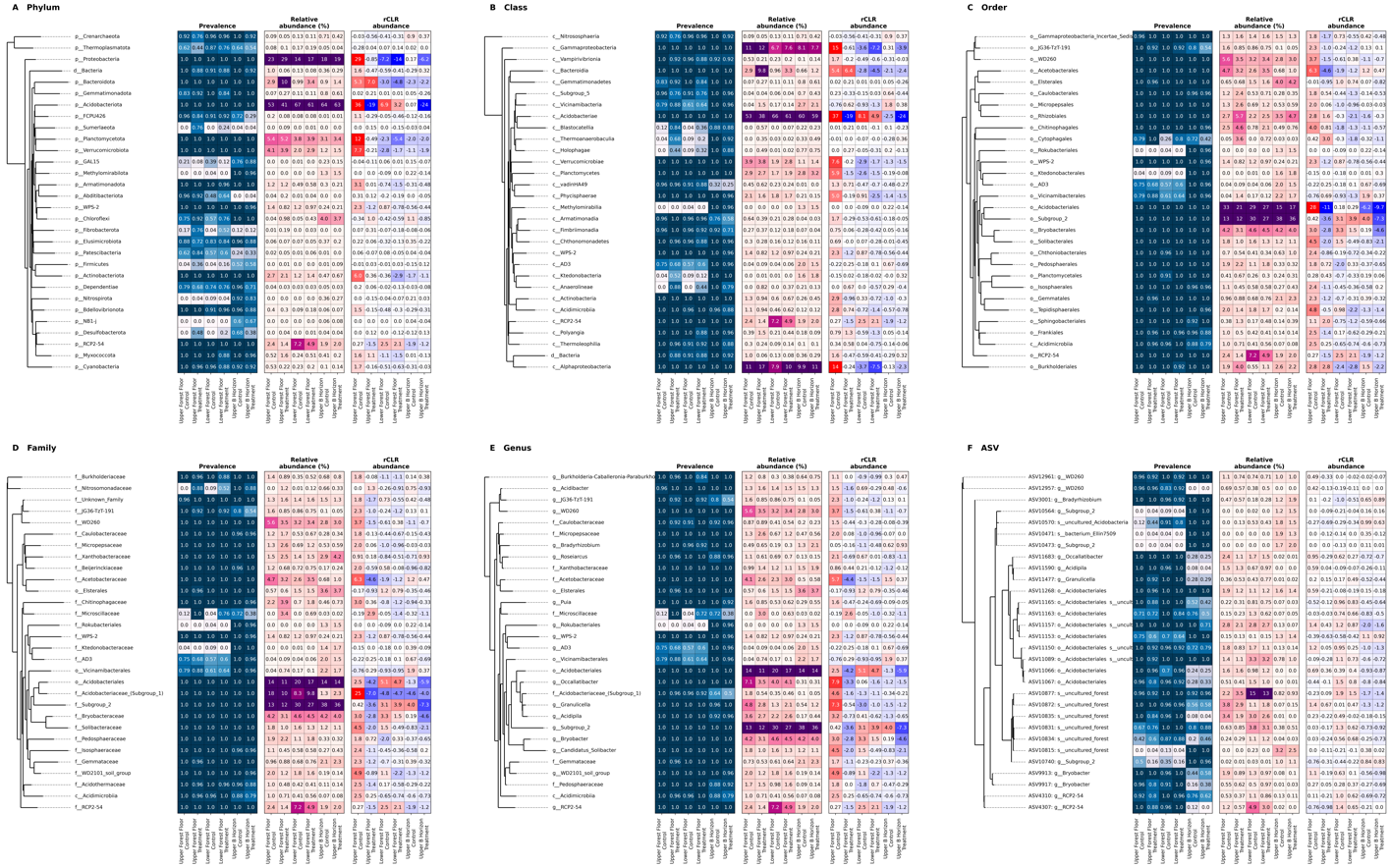**  **Supp. Figure 8 Heatmaps of the prevalence, relative abundance and rCLR abundance of prokaryotic taxa across sample groups**  The top 30 prokaryotic taxa identified within each of five phylogenetic levels (phylum, class, order, family, genus) and at the ASV levels are presented. The prevalence, percent relative abundance and robust central log ratio (rCLR) abundance are presented. The samples are grouped by control vs treatment and horizon. The boxes are colored based on metric (i.e. prevalence, percent relative abundance, rCLR abundance). |
| --- |

| **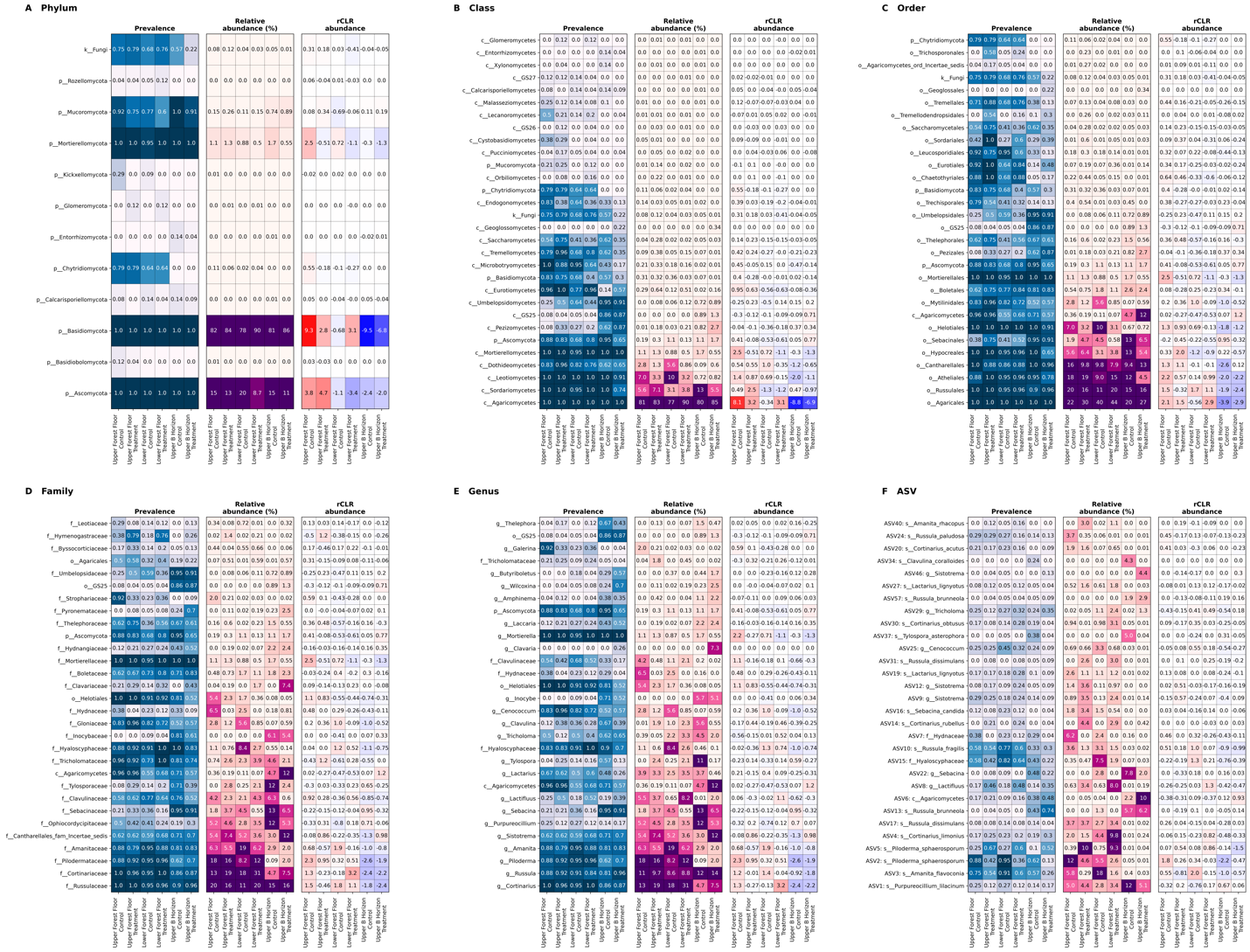**  **Supp. Figure 9: Heatmaps of the prevalence, relative abundance and rCLR of fungal taxa across sample groups**  The top 30 fungal taxa identified within each of five phylogenetic levels (phylum, class, order, family, genus) and at the ASV levels are presented. In some taxonomic level, there were not 30 taxa identified, in which case all identified taxa are presented. The prevalence, percent relative abundance and robust central log ratio (rCLR) abundance are presented. The samples are grouped by control vs treatment and horizon. The boxes are colored based on metric (i.e. prevalence, percent relative abundance, rCLR abundance). |
| --- |

| 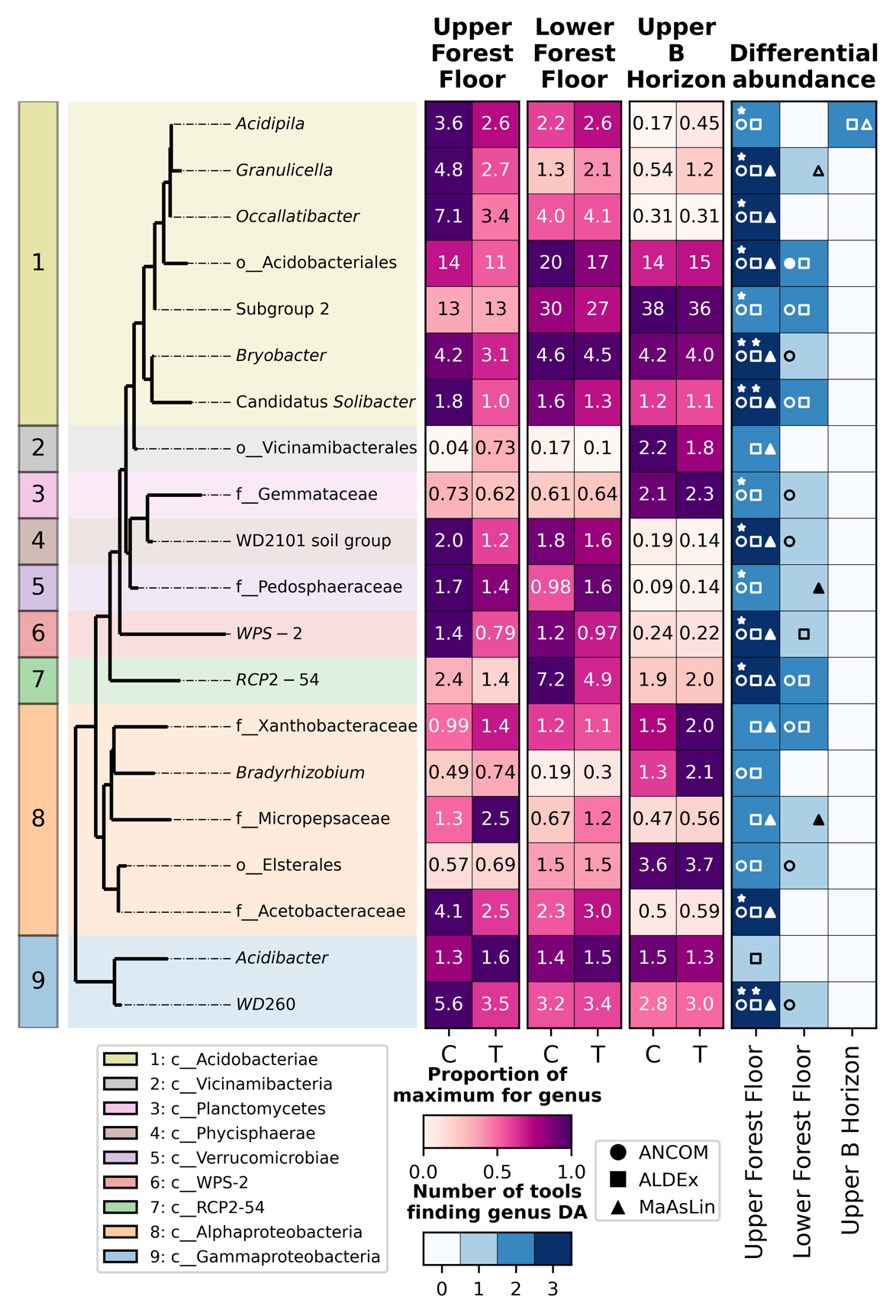 |
| --- |
| **Supp. Figure 10: The top 20 most abundant prokaryotic (16S rRNA gene) genera (relative abundance) DA testing including sampling round.** A phylogenetic tree where genera are coloured by the class that they belong to (or the lowest taxonomic level classified) (left). Heatmaps show the relative abundance of the genera of interest within each horizon + treatment group (middle). Values shown in the cells indicate the mean value for each soil horizon + treatment group. The results of the differential abundance tests (right) shows which of the tests (ANCOM, MaAsLin or ALDEx) found a genus to be significantly differentially abundant (indicated with markers) between control and treatment within each horizon. The boxes are colored by the number of tests that found it significantly differentially abundant. A filled shape indicates that the taxa remained significant when sampling round was considered. A star above the marker indicates a significant interaction between treatment and sampling round. Note that the interaction between treatment and sampling round was only considered for ANCOM and ALDEx; MaAsLin does not incorporate functionality for considering interactions between variables. |
| 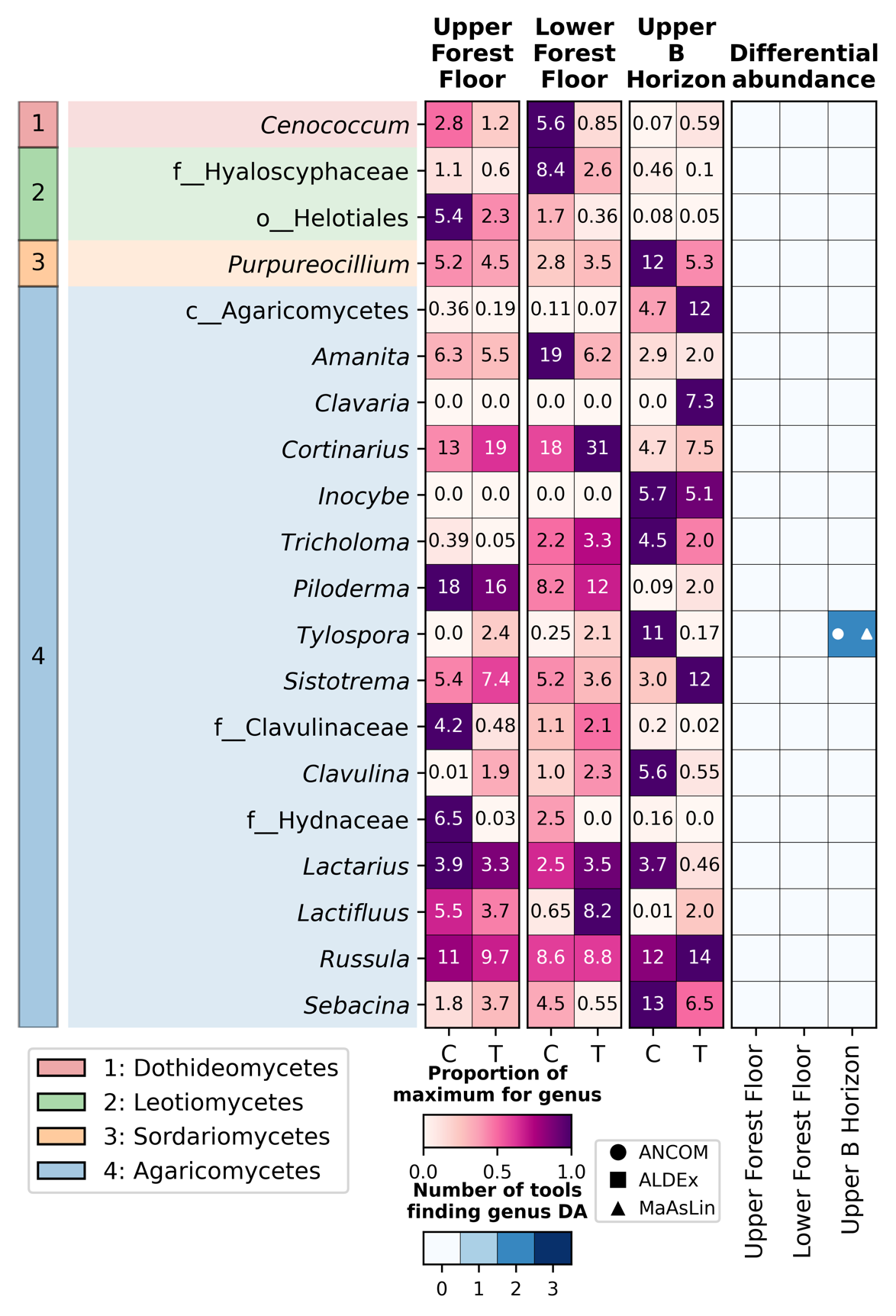 |
| **Supp. Figure 11: The top 20 most abundant fungal (ITS2 gene) genera (relative abundance) DA testing including sampling round.** A phylogenetic tree where genera are coloured by the class that they belong to (or the lowest taxonomic level classified) (left). Heatmaps show the relative abundance of the genera of interest within each horizon + treatment group (middle). Values shown in the cells indicate the mean value for each soil horizon + treatment group. The results of the differential abundance tests (right) shows which of the tests (ANCOM, MaAsLin or ALDEx) found a genus to be significantly differentially abundant (indicated with markers) between control and treatment within each horizon. The boxes are colored by the number of tests that found it significantly differentially abundant. A filled shape indicates that the taxa remained significant when sampling round was considered. |

Additional files submitted: Supplemental Table 1, Supplemental Tables 3&4, Supplemental Table 5, Supplemental Table 6, Supplemental Table 7, Supplemental Table 8, Supplemental Table 9

**Supplementary Table 1: Pre- and post-treatment** **soil chemistry data for the treatment and control plots.** The soil chemistry data collected from Otter Ponds for the twelve metrics (pH, calcium, magnesium, aluminum, manganese, organic matter, total carbon, total nitrogen, nitrate, ammonium, total sulfur, and sulfate) presented in Figure 2. The location of each plot is listed. There was a single sample from each horizon from each plot for pre-treatment sampling presented in the table. There were three samples from each plot from each horizon for post-treatment sampling and the average and standard deviations are presented in the table.

**Supplementary Table 3: PERMANOVA statistical tests for the 16S dataset**. The R^2^, p-values and adjusted p-values from the adonis test are presented for the various beta diversity metrics used.

**Supplementary Table 4: PERMANOVA statistical tests for the ITS2 dataset**. The R^2^, p-values and adjusted p-values from the adonis test are presented for the various beta diversity metrics used. Two of the beta-diversity metrics used the genus classification level versus species.

**Supplementary Table 5: 16S relative abundance at all taxonomic levels.** The relative abundance of 16S taxa collapsed to the taxonomic levels and ASVs are presented across all individual samples.

**Supplementary Table 6: ITS2 relative abundance at all taxonomic levels.** The relative abundance of ITS2 taxa collapsed to the taxonomic levels and ASVs are presented across all individual samples.

**Supplementary Table 7: Differential abundance test results for treatment.** The results of the three differential abundance tests (ALDEx, ANCOM and MaAsLin) for control vs treatment within each of the three horizons (upper forest floor, lower forest floor and upper B horizon) are presented for both the 16S and ITS data.

**Supplementary Table 8: Differences in abundance between control and treatment for differentially abundant 16S and ITS2 taxa.** The relative abundance and log2 relative abundance for 16S and ITS2 found differentially abundant (by 2 or more tests) are compared between control and treatment.

**Supplemental Table 9: Differential abundance test results for sampling round (timepoint) and interaction of treatment and sampling round.** The results of the three differential abundance tests (ALDEx, ANCOM and MaAsLin) for the two sampling rounds within each of the three horizons (upper forest floor, lower forest floor and upper B horizon) are presented for both the 16S and ITS data. The interaction between treatment (control vs treatment) and sampling round within each horizon is also presented.
